# Skeeter Buster: A Stochastic, Spatially Explicit Modeling Tool for Studying *Aedes aegypti* Population Replacement and Population Suppression Strategies

**DOI:** 10.1371/journal.pntd.0000508

**Published:** 2009-09-01

**Authors:** Krisztian Magori, Mathieu Legros, Molly E. Puente, Dana A. Focks, Thomas W. Scott, Alun L. Lloyd, Fred Gould

**Affiliations:** 1 Department of Entomology, North Carolina State University, Raleigh, North Carolina, United States of America; 2 Infectious Disease Analysis, Gainesville, Florida, United States of America; 3 Department of Entomology, University of California, Davis, California, United States of America; 4 Department of Mathematics and Biomathematics Graduate Program, North Carolina State University, Raleigh, North Carolina, United States of America; Mahidol University, Thailand

## Abstract

**Background:**

Dengue is the most important mosquito-borne viral disease affecting humans. The only prevention measure currently available is the control of its vectors, primarily *Aedes aegypti*. Recent advances in genetic engineering have opened the possibility for a new range of control strategies based on genetically modified mosquitoes. Assessing the potential efficacy of genetic (and conventional) strategies requires the availability of modeling tools that accurately describe the dynamics and genetics of *Ae. aegypti* populations.

**Methodology/Principal findings:**

We describe in this paper a new modeling tool of *Ae. aegypti* population dynamics and genetics named Skeeter Buster. This model operates at the scale of individual water-filled containers for immature stages and individual properties (houses) for adults. The biology of cohorts of mosquitoes is modeled based on the algorithms used in the non-spatial Container Inhabiting Mosquitoes Simulation Model (CIMSiM). Additional features incorporated into Skeeter Buster include stochasticity, spatial structure and detailed population genetics. We observe that the stochastic modeling of individual containers in Skeeter Buster is associated with a strongly reduced temporal variation in stage-specific population densities. We show that heterogeneity in container composition of individual properties has a major impact on spatial heterogeneity in population density between properties. We detail how adult dispersal reduces this spatial heterogeneity. Finally, we present the predicted genetic structure of the population by calculating F_ST_ values and isolation by distance patterns, and examine the effects of adult dispersal and container movement between properties.

**Conclusions/Significance:**

We demonstrate that the incorporated stochasticity and level of spatial detail have major impacts on the simulated population dynamics, which could potentially impact predictions in terms of control measures. The capacity to describe population genetics confers the ability to model the outcome of genetic control methods. Skeeter Buster is therefore an important tool to model *Ae. aegypti* populations and the outcome of vector control measures.

## Author Summary

Dengue is a viral disease that affects approximately 50 million people annually, and is estimated to result in 12,500 fatalities. Dengue viruses are vectored by mosquitoes, predominantly by the species *Aedes aegypti*. Because there is currently no vaccine or specific treatment, the only available strategy to reduce dengue transmission is to control the populations of these mosquitoes. This can be achieved by traditional approaches such as insecticides, or by recently developed genetic methods that propose the release of mosquitoes genetically engineered to be unable to transmit dengue viruses. The expected outcome of different control strategies can be compared by simulating the population dynamics and genetics of mosquitoes at a given location. Development of optimal control strategies can then be guided by the modeling approach. To that end, we introduce a new modeling tool called Skeeter Buster. This model describes the dynamics and the genetics of *Ae. aegypti* populations at a very fine scale, simulating the contents of individual houses, and even the individual water-holding containers in which mosquito larvae reside. Skeeter Buster can be used to compare the predicted outcomes of multiple control strategies, traditional or genetic, making it an important tool in the fight against dengue.

## Introduction

Mosquito-borne dengue virus serotypes cause approximately 50 million cases of dengue fever per year, 500,000 cases of dengue hemorrhagic fever (DHF) or dengue shock syndrome (DSS), and result in approximately 12,500 fatalities annually [1],[2]. Since the 1950s, the incidence of DHF/DSS has increased over 500-fold [2], due to increases in human population, uncontrolled urbanization and international travel [3]. The major vector for dengue is the mosquito *Aedes aegypti* which thrives in households with open, water-filled containers in which larvae develop. Lack of reliable piped water service and garbage disposal systems in endemic subtropical and tropical countries provide mosquito vectors with ample development sites [4].

Presently, there is no commercially available clinical cure for dengue and no vaccine has successfully completed clinical trials [5], leaving vector control as the only viable option for dengue prevention. Several practices are used to control dengue vector populations, including reduction or elimination of larval development sites and insecticides targeting immatures or adults. In the case of *Ae. aegypti*, the Container Inhabiting Mosquito Simulation Model (CIMSiM) [6],[7] is the most detailed tool available for understanding population dynamics and the expected effects of different intervention strategies on adult female densities.

CIMSiM is a weather-driven, dynamic life table simulation model of *Ae. aegypti* populations that incorporates a high level of detail about the life history of this species. Results from CIMSiM are used as the entomological input of a companion model, DENSiM [8], that models dengue transmission dynamics based on the mosquito population dynamics simulated by CIMSiM. CIMSiM and DENSiM have proven useful in characterizing local *Aedes aegypti* population dynamics [9] and predicting general impacts of control measures on dengue prevalence and incidence [10],[11]. Despite its considerable detail, three things that CIMSiM does not take into account are spatial heterogeneity in habitat availability, potential impacts of stochastic effects – both of which could significantly affect population dynamics – and the genetics of the simulated population. A stochastic spatial model of *Ae. aegypti* population dynamics has also been developed separately [12],[13] that does not include any genetic component.

A lack of a genetic modelling is not critical when dealing with most conventional methods of vector control unless evolution of insecticide resistance is of concern. Recent advances in molecular biology and genetic engineering, however, have presented the possibility of employing a number of control methods based on genetically engineered mosquitoes [14]. Genetic strategies fall into two broad categories: population suppression and population replacement. Population suppression methods, such as the Release of Insects carrying a Dominant Lethal (RIDL), which is a form of the sterile insect technique [15],[16], aim to reduce the density of vectors by releasing genetically engineered male mosquitoes that mate with native females and cause mortality of offspring before they emerge as adults. Population replacement strategies aim to replace the resident, competent vector population with mosquitoes that are genetically engineered to not transmit a pathogen [17]–[19]. For both approaches a model that can predict the outcome of releasing an engineered strain in a given location and across different ecological and epidemiological circumstances is critically needed to provide guidance for which particular approach (or combination thereof) would be the most effective and to anticipate any undesirable outcome.

To address this need, we developed a modeling tool, Skeeter Buster, that can predict how *Ae. aegypti* population dynamics and population genetics might be affected by stochasticity and spatial variation in *Ae. aegypti* habitat. Skeeter Buster builds on the biologically rich components of CIMSiM, while adding stochasticity, explicit spatial structure and genetics. The construction of this model is the first step of our project that aims at evaluating conventional and genetic vector management tools and their potential success in controlling dengue incidence in human populations. To that end, Skeeter Buster will ultimately be associated with an epidemiological model. This tandem modeling tool will be comparable to the CIMSiM/DENSiM association, and will allow a direct assessment of the effects of vector control measures (including genetic approaches) on dengue prevalence and incidence. The latest version of Skeeter Buster with a user-friendly interface is available for Windows platforms at http://www.skeeterbuster.net, and the source code is available on request from the authors.

In this first paper, we explain the characteristics and specificities of Skeeter Buster, and present results from simulations that compare the population dynamics predictions of Skeeter Buster to those of CIMSiM. Examples presented are not intended to explore the vast parameter space that is associated with this model. All parameters are, however, adjustable in the user-friendly version of the modeling tool. Subsequent articles will describe a detailed sensitivity and uncertainty analysis of this model, as well as validation against a data set of *Ae. aegypti* population dynamics in Iquitos, Peru. These detailed analyses will indicate whether there are specific details in the model that are not important for predicting the dynamics of *Ae. aegypti*, and could be dropped from the model. These analyses may also point to specific parameters in the model that have major effects on the mosquito dynamics, and therefore require better empirical estimates.

## Methods

### General characteristics of CIMSiM and Skeeter Buster

**Figure 1:**
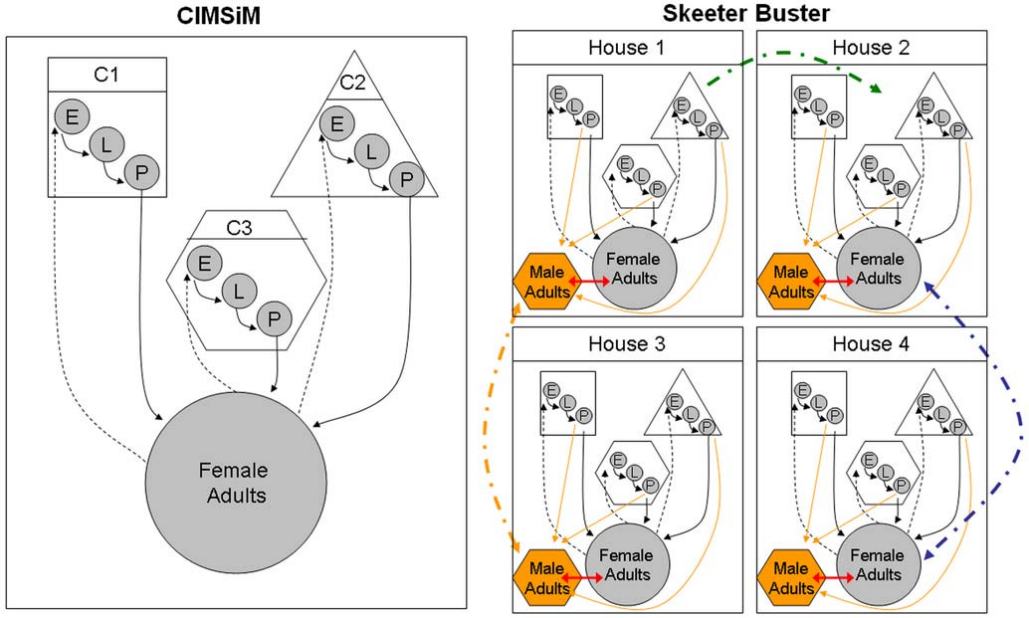
Schematic representation of the general structure of CIMSiM and Skeeter Buster. C1, C2 and C3 are representative containers of three different types. E: eggs – L: larvae – P: pupae. Solid arrows represent transition of cohorts between life stages. Dashed arrows represent oviposition. Grayscale items represent model parts that are identical in CIMSiM and Skeeter Buster. Colored items are specific to Skeeter Buster. Multiple properties are modeled in Skeeter Buster. Only 4 properties are represented in this schematic figure, but the number of simulated properties can be (and typically is) much higher. Orange boxes and lines represent male adults and their biology. Red arrows represent mating, which is restricted to individuals present at the same property. Dash-dotted orange and blue lines represent male and female dispersal, respectively. Although dispersal may occur between all neighboring properties, to improve clarity it is only depicted between properties 1 and 3 for males, and between properties 2 and 4 for females. Dash-dotted green line represents container displacement. Although displacement may occur for all containers and towards any property, for clarity it is depicted only once.

Because Skeeter Buster was built using many algorithms from CIMSiM, the two models share a number of identical characteristics. We schematically describe the relationships between the two models, with identical components represented in grayscale and specific additions in Skeeter Buster in color (Figure 1).

CIMSiM is a deterministic, weather-driven model that follows cohorts on a daily time scale for each immature stage (eggs, larvae, pupae) as well as female adults. Because they are not considered to impact the population dynamics, adult males are not modeled in CIMSiM. Environmental parameters include daily weather data (temperature, precipitation, and relative humidity) and an external input of food into containers. Based on these variables, CIMSiM calculates the number of individuals within all cohorts present in the model at a given time, their cumulative physiological development, weight, fecundity and gonotrophic status, as well as the transitions between life stages. With the exception of food input, all calculations in CIMSiM are applied to cohorts in a strictly deterministic fashion.

CIMSiM calculates water temperature and water level in all containers based on the available local weather data. The amount of food available in each container is also calculated daily. In addition to food depletion by consumption, three factors affect the amount of food: the external food input, a daily decay factor and the conversion of dead immatures to nutritional resources. The daily survival probability for each life stage includes a temperature-dependent component. Cumulative physiological development of each life stage is also based on temperature using an enzyme kinetics approach [20] assuming that a single enzyme determines the development rate of the insect (see equations in Text S2.2). Completion of physiological development at a given stage is attained when cumulative development reaches a threshold value (specific for each life stage). Hatch of embryonated eggs is determined by water level and water temperature in the container. Larval weight is modeled in parallel with the amount of food in each container according to the equations in [21](see in Text S2.4). Pupation requires larvae to complete physiological development as well as reach a sufficient weight. Fecundity of female adults is based on their weight, and females distribute their eggs among available containers based on the size of these containers.

These general characteristics of CIMSiM are all incorporated into Skeeter Buster, but with three major differences. First, Skeeter Buster is a stochastic model. For a given event (*e.g.* survival) applied to a specific cohort, a probability is defined for the cohort, and that same probability is applied independently to all individuals within the cohort. The number of individuals to which the event occurs is obtained by drawing a number from a binomial distribution defined by that probability and the total number of individuals in the cohort. Second, Skeeter Buster models several distinct locations (hereafter called “properties”). In the simplest setup, properties are arranged on a rectangular grid, and sets of distinct water-holding containers are assigned to individual properties (indoor or outdoor location of each container is specified). Immature cohorts are associated with a specific container within a property, and emerging adults are associated with a specific property. Finally, because Skeeter Buster also models the genetics of the population, cohorts are further distinguished by genotype.

Skeeter Buster also includes a number of components lacking in CIMSiM (see Figure 1). First, because of the genetic component of Skeeter Buster, male adults are now included in the model. Consequently, an important new component is the modeling of mating in the population. Mating is restricted to individuals present at the same property. Adults can disperse from one property to another, and containers can also be transported between properties, with the assumption that egg cohorts are carried along in the container.

In the following sections, we describe the Skeeter Buster model in more detail. We first describe the dynamics within a single property and within individual containers, and then describe the spatial structure of the model and mosquito movement among properties. We provide a complete description of the processes involved in Skeeter Buster. Some of these processes are similar to those in CIMSiM and are described in [6]. Therefore, we only describe those processes briefly in the main text, and refer the reader to supporting material (Text S2, Text S4, Dataset S1) for more details about the equations and parameters that are identical to their equivalent in CIMSiM.

### Local population dynamics of immature and adult *Ae. aegypti*

#### Eggs

The number of *Ae. aegypti* eggs surviving per day is determined by the current water and air temperatures, sun exposure, and water depth of the breeding container (see Fig. 3 and 4 in [6], and Text S2.7 and S2.8).

**Figure 2:**
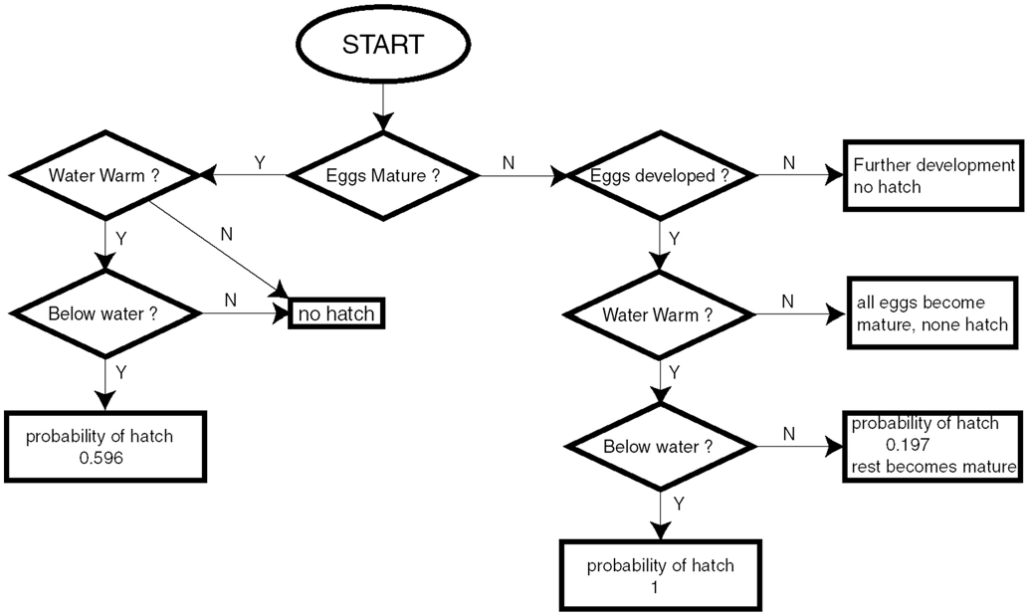
Algorithm for the determination of egg hatch probabilities. Process flow diagram representing the algorithm that is followed each day to determine the hatch probability of *Ae. aegypti* eggs in a cohort.

Egg hatch is one of the most intriguing and complicated parts of the biology of *Ae. aegypti* [22]. We illustrate how Skeeter Buster (based on CIMSiM) determines the number of eggs hatching daily in a specific breeding site (Figure 2), which is only described briefly in [6]. Freshly laid eggs have to first develop sufficiently to finish embryonation. The rate of physiological development of eggs to embryonation depends on the average water temperature (parameters are taken from [6], see Text S2.2) and development accumulates until embryonation is finished. Embryonated eggs have to fulfill two additional requirements to hatch: the average water temperature has to be above 22°C, and the eggs have to be submerged in water. If the water is warm enough and the eggs are submerged, all newly embryonated eggs hatch immediately. If the average water temperature is below 22°C when the eggs finish embryonation, none of them hatch, and they enter a “mature” state. If the water is warm enough but the eggs are not submerged, a certain proportion (19.7%) of the eggs still hatch [23], and it is assumed that those larvae drop into the water, while the remaining eggs enter the “mature” state. 59.6% of “mature eggs” hatch every day when they are submerged in water with an average temperature above 22°C. Without submergence, none of these eggs hatch.

Eggs that hatch transform into neonate larvae in the container. Neonate larvae that hatch in the same container on the same day are separated into new larval cohorts with unique genotypes (including sex). Initial weight of neonate larvae is assumed to be 0.001 mg [21] as opposed to 0.0034 mg used in CIMSiM.

#### Larvae

We do not model different larval instars separately. Instead, we track larval weight, fat reserves and cumulative physiological development. In order to pupate, larvae need to meet two criteria. First, larvae need to reach complete physiological maturity, *i.e.* the cumulative physiological development has to exceed a certain threshold. We call larvae that have met this criterion “developed”. Then developed larvae pupate only if they have reached sufficient weight. The weight threshold that a developed larval cohort has to exceed in order to pupate is itself dependent on the cumulative physiological development of the cohort. The weight threshold is lower for physiologically older larvae. We describe first how physiological development and larval weight are modeled, then how these two parameters define the criteria for pupation. Finally we describe survival algorithms for larval cohorts.

#### Larval physiological development and maturation

In CIMSiM, all larvae from a cohort become developed on the same day that their cumulative physiological development reaches 0.95 or more. However, in Skeeter Buster we introduce more realistic variability in the date of development completion by allowing some portions of the larval cohort to become developed at a lower cumulative physiological development, and other portions to reach more than the mean physiological date of maturation before they become developed. We assume that the probability of becoming developed for an individual larva is a function of its cumulative physiological development, with no larvae becoming developed below a cumulative physiological development of 0.89 and all larvae becoming developed above 1.17 [24]. In between these two extremes, each larva becomes developed with a probability based on the cumulative physiological development of the larval cohort (see equation in Text S2.3 and Figure S3). Developed larvae from each larval cohort are moved to a newly created cohort to avoid coexistence of developed and undeveloped larvae in the same cohort. This does not significantly increase the number of larval cohorts in the model at any given time because larval physiological development completion is typically spread only over two days.

#### Larval weight, food calculations and density-dependence

As in CIMSiM, the dynamics of larval cohort weight and the amount of larval food in a container are governed by equations based on a laboratory study of larval development [21] (see Text S2.4). Dry weights of larvae and pupae are used here in accordance with the equations in [21]. The equations determine the change of larval weights and food levels during 4 hrs intervals, which are integrated by the Euler method for 24 hrs. The original equations in [21] were designed for and validated at 26°C. The equations are supplemented in CIMSiM and Skeeter Buster by the addition of a factor that scales for different temperatures [6]. All members of a larval cohort grow in weight uniformly, and male and female larvae grow at the same rate under identical conditions.

**Figure 3:**
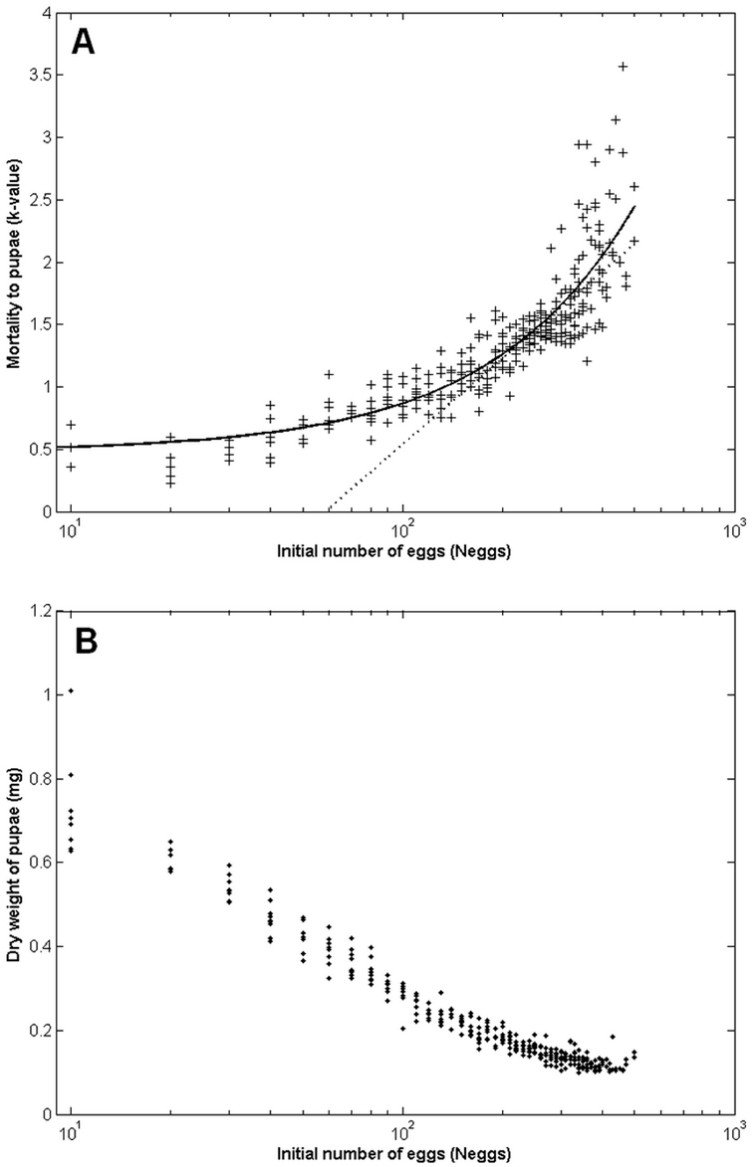
Density-dependence within a typical container (1-gallon bucket) in Skeeter Buster. The development of a single mature egg cohort (with *N_eggs_* eggs) is tracked in a single container (10 replicated simulations). A: mortality from egg to pupa, expressed as a *k*-value: if *N*_pup_ is the final number of surviving pupae, *k* = −log(*N*_pup_/*N*_eggs_). ‘+’ symbols represent the outcomes of individual simulations. Solid line is the fit of the model *N*_pup_ = λ. *N*_eggs_.exp(−α*N*_eggs_^β^) [26]. Dashed line represents the point at which the slope of the curve is exactly one (*N*_eggs_* = 253), marking the transition from undercompensatory (slope<1) to overcompensatory (slope>1) density-dependence (see text). B: average dry weight (in mg) of the surviving pupae.

These equations describing the dynamics of larval weight and available food govern the density-dependent competition for food among conspecific larvae within containers. Density-dependence is considered a major component of larval dynamics in container breeding mosquitoes such as *Ae. aegypti* [25], and is suspected to be mostly caused by indirect competition for nutritional resources. Few studies have provided a detailed description of density-dependence for *Ae. aegypti* [23],[26], yet the details of these dynamics are critically important for vector control purposes. The degree and pattern of density-dependence in natural populations is not well known [27]. We present a simple example of the type of density-dependence existing in Skeeter Buster, showing a major impact on two important immature traits: the survival from egg to pupa, and the weight of the surviving pupae (Figure 3). By fitting a simple model of density-dependence [26] to these results, we can distinguish how density-dependence affects survival to pupae depending on the initial density (Figure 3A): at low initial densities, undercompensatory density-dependence is observed (*i.e.* an increase in initial density results in an increase in the number of survivors to pupa), while overcompensatory density-dependence is observed at higher initial densities (*i.e.* an increase in initial density results in a decrease in the number of survivors to pupa). Note that this illustration of density-dependent processes within a single container ignores interactions between early and late instars from unsynchronized cohorts, and therefore probably underestimates the effects of density-dependence.

#### Larval survival

Temperature-dependent larval mortality is determined according to Fig. 3 in [6] (see also Text S2.7) based on the minimum and maximum water temperature. Larvae die with a probability of 0.95 per day if the container dries up.

**Figure 4:**
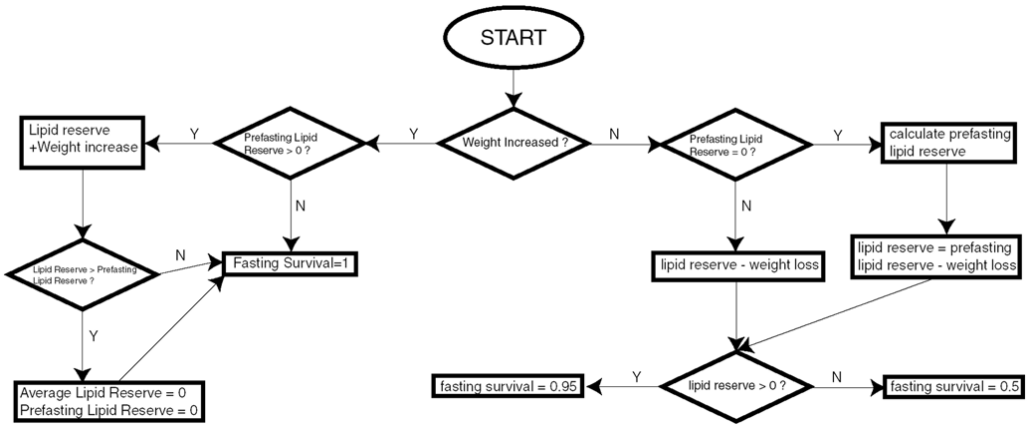
Algorithm for larval survival probability calculations. Flow diagram depicting the algorithm that determines the daily survival probability of *Ae. aegypti* larvae that are currently experiencing or recovering from starvation. PLR = Prefasting Lipid Reserve.

The most complex cause of mortality is starvation. Figure 4 illustrates the process of determining starvation survival. The definition of starvation is that the weight of the larvae in the cohort decreases from the previous to the current day. On the first day of starvation, a prefasting lipid reserve for the larval cohort is calculated, based on Eq. 9 and 10 from [21]. During starvation, the actual lipid reserves of the larvae are reduced by the weight lost, which in turn, impact larval fasting survival. Starving larvae that still retain lipid reserves have an additional 0.05 probability of mortality compared to non-starving larvae, whereas those without reserves have an additional 0.5 probability of mortality per day. Larvae that have been starving previously but are now regaining weight increase their lipid reserves and have no additional mortality due to starvation. Whenever the amount of actual lipid reserves of a larval cohort that has been previously starving reaches its prefasting lipid reserve, the starvation period is considered to be over.

All larvae in a cohort die if they accrue a cumulative physiological development above a maximum threshold (set at 800% of the mean development period) and still have not gained enough weight to pupate. This prevents larvae from lingering in poor nutrient conditions indefinitely. Similarly, all larvae in a cohort die if their weight drops below a specific threshold for survival (arbitrarily set to 90% of the weight at hatching, *i.e.* 0.0009 mg). Dead larvae are converted into biomass for larval food on the next day, with a 0.4 conversion factor.

#### Pupation

Larval pupation is modeled as in CIMSiM, where developed larvae have to reach a pupation weight threshold, which is a decreasing function of both larval cumulative physiological development and temperature. The progression of larvae in larval cohorts in terms of their cumulative physiological development as well as their weight is illustrated by their growth trajectories (see Figure S4). In CIMSiM, pupation weight thresholds are identical for males and females, and the entire larval cohort pupates together when its members reach the pupation weight threshold. In Skeeter Buster, pupation weight thresholds are lower for males compared to females, which is more realistic [28]. In addition, we created separate pupation weight thresholds at which the probabilities of pupation for members of the larval cohort are 0.25, 0.5, 0.75 and 1, respectively. The pupation weight threshold calculated in CIMSiM represents the value at which this probability equals 0.5. In CIMSiM, the pupation weight threshold is based on the cumulative physiological development of the larval cohort on the previous day. In Skeeter Buster, it is based on the cumulative physiological development calculated on the current day. Larvae entering pupation die with a probability of 0.05 due to developmental abnormalities [19] and are converted to larval food with 0.4 conversion rate. The remaining pupating larvae are assumed to successfully enter the pupal stage. In Skeeter Buster, the dry weight of members of a new pupal cohort is equivalent to the dry weight of individuals in the mature larval cohort from which those pupae originated. Therefore, if multiple larval cohorts pupate in the same container during a given day, the pupae originating from each larval cohort will be transferred into corresponding separate pupal cohorts. In contrast, CIMSiM merges pupae originating from multiple larval cohorts into a joint pupal cohort with dry weight of the new pupae calculated as the average of the dry weights of the multiple larval cohorts. Skeeter Buster hence preserves the heterogeneity in pupal weight, and ultimately adult weight, which is a trait of epidemiological importance [29].

#### Pupae

Because mosquito pupae do not feed, completion of this developmental stage, and emergence of adults, occurs as soon as pupae reach physiological maturation. In CIMSiM, all pupae in a cohort mature on the same day when their cumulative physiological development reaches 0.95 or more. In Skeeter Buster, we assume that the probability of maturation for an individual pupa is a function of its cumulative physiological development, as described above for larvae. No pupa matures below the cumulative physiological development of 0.89 while all pupae mature above 1.17 [24]. In between these two extremes, each pupa in the cohort becomes mature with a probability based on the current cumulative physiological development of the cohort (see Text S2.3). If any of the pupae mature, a new “mature” pupal cohort is created in order to store them separately. This does not increase the number of necessary pupal cohorts significantly because pupal physiological maturation is typically only spread over two days.

Pupal survival in Skeeter Buster depends on water temperature according to Fig. 3 in [6]. Dead pupae are converted into biomass for larval food on the next day, with a 0.4 conversion rate. All surviving pupae in a mature pupal cohort emerge on the same day. Significant mortality (17%) of pupae during eclosion is assumed as in CIMSiM. Unlike CIMSiM, Skeeter Buster accounts for pupae dying during eclosion as a source of biomass for larval food.

#### Adults

Pupae that successfully eclose become nulliparous female or male adults. In CIMSiM, all female adults that eclose on the same day from multiple containers form a new single female adult cohort. The adult weight associated with this cohort is equal to the average weight of all contributing pupal cohorts multiplied by a conversion factor from dry weight used in larval/pupal calculations to wet weight used in adults. Therefore, small females that eclose from a suboptimal container and large females that eclose from a very productive container on the same day are merged into a joint cohort of average-sized females, for computational simplicity. Given the importance of the size of females for traits such as fecundity [30] and blood-feeding frequency [29],[31], Skeeter Buster separately treats each pupal cohort from which new adults eclose. Emerging adult females are modeled individually (so that they can later be tracked in the epidemiological model), while emerging males from each pupal cohort are transferred to a new male adult cohort. The weight of newly eclosed adults is the weight of the pupae they eclosed from, converted from dry weight to wet weight.

Because the goals of CIMSiM focus on population dynamics, and males are not generally considered to contribute to the population dynamics of *Ae. aegypti*, adult males emerging from containers are discarded in CIMSiM. In contrast, a major goal of Skeeter Buster is to assess the outcome of genetic control strategies, for which males are critical, and therefore included in the model. CIMSiM also assumes that the sex ratio of emerging pupal cohorts is always exactly 1∶1. However, there is compelling evidence that in stressed conditions the sex ratio of emerging pupal cohorts is significantly skewed in the direction of males, in extreme cases leading to emergence of only males [32]. Skeeter Buster can reproduce such patterns by the assumption of a lower pupation weight threshold for males than for females. In some resource-limited settings, the majority of male larvae can reach their pupation weight threshold and successfully pupate and eclose, while many female larvae can fail to reach their higher pupation weight thresholds and die. Such a skewed sex-ratio would affect the population dynamics and genetics of the mosquito population, which might have particularly important consequences for genetic control strategies.

Daily mortalities of male and female adult mosquitoes are likely to be extremely variable and change with local conditions. We assume nominal daily mortality probabillities of female and male adults to be 0.11 and 0.23 respectively, based on field estimates [33], as opposed to 0.09 for both sexes assumed in CIMSiM. The daily mortality probability of adult mosquitoes also depends on the minimum and maximum air temperature and on saturation deficit (see Text S2.7 and S2.8). Additionally, age-dependent mortality can be set to occur in Skeeter Buster by setting an age at which senescence starts and the maximum age that an adult male or female can reach (see e.g. [32]). Between these two ages, daily mortality probability increases linearly from the base value at the onset of senescence up to 1.0 at the maximum age.

The status of female adults in the gonotrophic cycle is defined by their cumulative physiological development and is modeled using an enzyme kinetics approach similar to the one described for immature stages. We assume that females are not limited by the availability of blood meals, that hosts are always available everywhere and are homogeneous in quality (details about blood feeding behavior will be accounted for in the future epidemiological model). Female adults are assumed to oviposit when they complete their gonotrophic cycle (*i.e.* when their cumulative physiological development reaches a threshold value). The first gonotrophic cycle is assumed to be considerably longer than the subsequent ones because all ovarioles of a female adult have to progress from Christopher's stage I to stage II during this cycle [34]. As with CIMSiM, Skeeter Buster accounts for this difference in cycle length by requiring that females complete 100% of cumulative physiological development during the first gonotrophic cycle, while in subsequent cycles, females only need to complete 58% of cumulative physiological development (assuming that 42% of development occurs before the cycle begins).

In CIMSiM, the number of eggs laid by female adults on a specific day is an increasing linear function of the moving average of the wet weights of the last five eclosing female adult cohorts. While this method simplifies the computation, the moving average may include the wet weight of female adult cohorts that do not complete their gonotrophic cycle on the current day or even cohorts that eclosed so long ago that the individuals they represent are already all dead. Additionally, the moving average in CIMSiM is not weighted according to the relative numbers of female adults in the respective female adult cohorts. In contrast, Skeeter Buster models female adults individually, and determines the mean fecundity of ovipositing females based on their unique wet weight. The number of eggs laid by each individual adult female in each cycle is determined randomly by drawing numbers from a normal distribution of values based on the mean fecundity, with a standard deviation of 0.375 times the mean [35]. When females are over 25 days old, their mean fecundity decreases linearly (−0.437 eggs per day over 25 [36]).

We assume that females are strictly monogamous, so that each female mates only once in its lifetime. Sperm received in this mating are then used to fertilize eggs in all subsequent gonotrophic cycles. The literature on the mating behavior of *Ae. aegypti* includes conflicting opinions on the ability and frequency of adult female *Aedes aegypti* to re-mate. While some of the sources suggest that adult females mate only once during their whole lifetime [37],[38], there is evidence to show that they mate at least once every four gonotrophic cycles [39],[40]. We assumed a single mating because only a small portion of adult females lives until the end of four gonotrophic cycles. However, if future studies of field populations report a significant amount of polyandry, the model could be modified to allow females to have multiple mates.

The list of male cohorts available for mating at each property is compiled daily. This only includes male adult cohorts that emerged more than 2 days ago, because adult males need 48 hrs for their external genitalia to turn 180° into the position needed for mating [22]. Male adult cohorts of the same genotype are merged in this list, so that the list of available male adult cohorts for mating only comprises one male mating cohort per genotype. Because there is evidence that male body size is positively correlated with reproductive success [41], we rank each male mating cohort according to the total wet weight of all adult male individuals of that genotype. At a specific property, the probability that a male in a mating cohort with a specific genotype is chosen for mating is based on the mating rank of that genotype, which is solely based on weight (we currently assume that genotype itself does not impact male mating ability).

In Skeeter Buster, genotypes are represented as a binary sequence of loci. We allow only two alleles at any given locus. The genotypes of the offspring resulting from the mating of a female and male of particular genotypes are determined by a random sampling of the possible gametes of the two parents.

Eggs laid by adult females of the same cohort at the same property on a single day are separated by genotype and distributed to all available containers at the property. Each container receives eggs with a probability proportional to the logarithm of the container volume. A given egg batch is distributed among containers using multinomial distributions based on these probabilities, with more eggs laid in larger containers than in smaller ones [42]–[44]. Eggs of the same genotype that are deposited into the same container (by all females at a given property) are summed to create a new egg cohort that is unique in terms of genotype and container location.

The exact height within the containers at which an egg is deposited is important in terms of survival and hatching. *Aedes aegypti* distributes its eggs on the sides of the containers within a band from the water level up to a few centimeters above the water level. While CIMSiM divides the height of containers into 2 cm layers, Skeeter Buster uses a finer 2 mm resolution, which conforms better to the natural size of mosquito eggs [22]. Egg cohorts are distributed into a maximum of 20 bands (*i.e.* 4 cm) above the water level, using a multinomial distribution with equal probabilities. If the water level is less than 4 cm below the top of the container, eggs are similarly distributed into the available number of bands between the water level and the top of the container.

### Spatial dynamics and movement among properties

While CIMSiM models a single representative area with a default size of 1 ha, Skeeter Buster models multiple properties independently. Each property hosts a specific set of containers both inside and outside of buildings, and the immature cohorts in these containers as well as the adults emerging from those are specifically assigned to that particular property. Properties are laid out on a rectangular grid, each cell of the grid representing a single property. The grid is not associated with explicit geographic distances, and the property is the only fundamental unit of distance. Although in this paper we consider properties to be at the scale of meters (individual houses in a dense urban setting), one property in the model can be considered to be larger units such as a block of properties or a village (and parameters can be adjusted accordingly) if needed for specific questions.

Properties located on the edges of the grid have fewer immediate neighbors than those in the interior of the grid. To deal with these edge locations we employ one of three boundary assumptions. First, periodic boundaries assume that opposite borders of the grid are connected to each other to form a toric topology. Second, solid boundaries prevent mosquitoes from migrating across the border, and force them to stay in the border property. Third, with random boundaries, mosquitoes migrating across a border are reintroduced at a random location on the edges of the grid. Properties can be identified by their coordinates (x_i_, y_i_) on the grid. Distance between properties (x_i_, y_i_) and (x_j_, y_j_) is defined as |x_j_−x_i_|+|y_j_−y_i_| (with appropriate adjustments depending on the boundary conditions). In this paper, we only report results from model runs that use solid boundary conditions.

**Figure 5:**
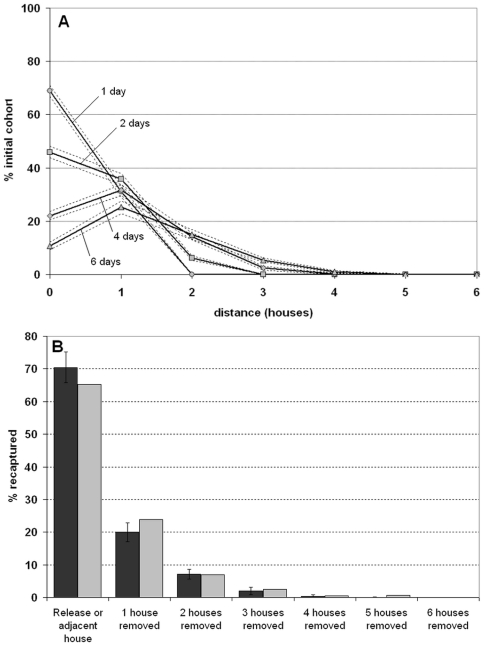
Dispersal of a single female adult cohort in Skeeter Buster, and virtual mark-recapture experiment. (A) Dispersal of a single female adult cohort released in a single property at day 0. Only short range dispersal is allowed (daily rate = 0.3), and survival is set at the default value (daily rate = 0.89). Solid lines represent the average number of females (20 replicated simulations) found at a given distance from the release house after 1 (circles), 2 (squares), 4 (diamonds) or 6 (triangles) days, dashed lines are 95% CIs. (B) Virtual mark-release-recapture (MRR) experiment based on this single cohort. We replicate the protocol and recapture rate described in Harrington *et al.* (2005) (Table 2, line 1) for outdoor releases in Thailand, with daily recaptures for 12 consecutive days. Dark bars are the results from the virtual MRR in Skeeter Buster (+/−SD), light bars are the results observed by Harrington *et al.* (2005).

Adults can disperse between properties. Skeeter Buster allows for both short and long range dispersal. Short range dispersal allows adult male and female mosquitoes to move to nearest neighbor properties. We assume that this movement occurs with probability 0.3 for each mosquito on each day. We estimated this probability by simulating an empirical mark-release-recapture study in Thailand [45], and measuring the necessary daily dispersal probability to match the distribution of captured marked mosquitoes found in that study (Figure 5). In the model, for each dispersing adult, one of four directions is randomly chosen, and the adult is moved to the nearest property in that direction (von Neumann neighborhood).

Adult mosquitoes can disperse to properties at a further distance in the grid by long range dispersal. There is no clear consensus in the literature about the extent of long range dispersal of adult *Ae. aegypti* (*e.g.* how often this happens, or how far adults migrate) [45]–[47]. In Skeeter Buster, each adult can disperse long distances with a daily probability; we assume a default value of 0.02. A maximum distance is also defined for long range dispersal events (default value of 20 properties, corresponding to ∼200 meters in a dense urban setting). Within this range, an actual distance is chosen at random (uniformly between 1 property and the maximum distance), and the destination property is chosen randomly among properties situated at this particular distance. We assume that the dispersal probabilities for both short and long range dispersal are independent of age, sex [45], parous state, mating status, size or developmental percentage.

Finally, we also allow the possibility for displacement of containers from one property to another. With some daily probability, any particular container is removed from its original property and allocated to another randomly chosen property in the grid. To account for the movement of immature cohorts associated with container displacement, all egg cohorts present in a moving container remain unaltered by this process. Larval and pupal cohorts, however, are discarded. In this paper, unless otherwise specified, the daily movement probability is assumed to equal zero.

### Simulation program development

To develop the Skeeter Buster simulation program, we chose to rewrite a clone of CIMSiM in C++ as a first basis, because it provides several clear advantages for model development. From a practical point of view, CIMSiM was originally written in Visual Basic, a coding language that is tied to the PC platform and that has undergone alterations that hinder recompilation of the code on recent machines. We instead chose to use standards-compliant C++ to provide maximum flexibility, e.g. in allowing the code to be ported to and run on other computer systems, and to prevent future obsolescence of the code.

Another, more important, motivation for our strategy was to provide some means to verify our simulation code, ensuring that all procedures would work in Skeeter Buster according to the algorithms presented in the original published model [6]. The complexity of the CIMSiM (or Skeeter Buster) simulation code offers many opportunities for the occurrence of coding errors; these could be difficult to identify without an independent rewrite of the code. Rewriting CIMSiM allowed us to reveal and correct some inconsistencies between the original model and presented algorithms, as well as apparent malfunctions in the original release of CIMSiM (see Text S1, Figure S1 and Figure S2). For all the above reasons, we felt that the rewriting process of CIMSiM was a necessary step prior to working with confidence when expanding the initial model to build Skeeter Buster.

**Figure 6:**
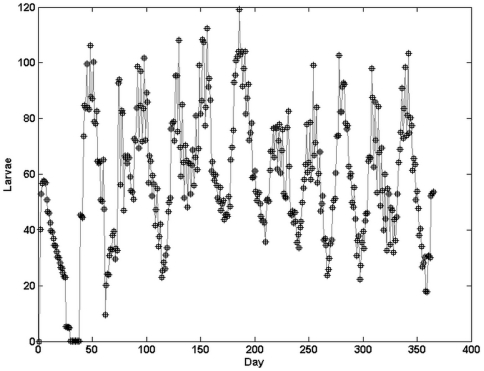
C++ CIMSiM as a clone of the original CIMSiM. Number of larvae generated by the original CIMSiM (squares) and the version of C++ CIMSiM (+signs) that incorporates the small errors and malfunctions detected in the original CIMSiM, showing perfect match. Weather data were collected in Iquitos, Peru during 1978. Containers used were 1 gallon buckets [6], and both models simulated an unstructured 1-ha area. All parameters were set as in [6].

We rewrote CIMSiM in C++ (hereafter refered to as C++ CIMSiM) by exactly following the algorithms described in [6]. We tested C++ CIMSiM by systematically comparing its output to the output of the original CIMSiM program with identical parameters. Whenever the output was different, we contrasted the source code of the C++ CIMSiM to the algorithms published in [6] as well as to the source code of the original CIMSiM. We corrected several coding errors in C++ CIMSiM (see Text S1). We observed several differences between the operation of the original CIMSiM program and the algorithms described in [6]. In order to verify C++ CIMSiM, we had to deliberately include these differences and coding errors from the original source code into the C++ CIMSiM source code during this testing phase. We attributed rare remaining differences in the outputs to malfunctions of the original CIMSiM executable. We were able to mimic such malfunctions by deliberately altering specific cohorts of larvae on specific occasions in C++ CIMSiM (see Text S1). Finally, we were able to match the output of the original CIMSiM executable and the output of C++ CIMSiM (Figure 6). Because C++ CIMSiM is not affected by the malfunctions in the original CIMSiM executable and is more flexible in terms of desired output, we used C++ CIMSiM in our comparisons to Skeeter Buster.

Skeeter Buster was developed by expanding and modifying this C++ code according to the model specificities described above (see Text S4 for a detailed list of modifications). A user-friendly graphical interface was developed for PC/Windows systems, and allows the user to vary parameter values. This part of the code is more specific to the particular system, but a similar interface could be developed for other systems (or could be developed in a portable framework such as Java).

## Results

In this paper we present results of Skeeter Buster simulations and compare them to output from CIMSiM (using our C++ CIMSiM version). The simulations presented here use weather data from the city of Iquitos, Peru, collected from the NCDC CDO online database [48]. Iquitos is a geographically isolated city in the Amazon basin whose *Ae. aegypti* population, larval habitat composition and dengue transmission dynamics have been studied for over 9 years [49]–[51]. In the simulations presented here we limited the properties to having only three types of containers: 1-gallon and 5-gallon plastic buckets, and 55-gallon drums. Detailed physical specifications of these containers are taken from [6]. These container types appear to be the two dominant types in Iquitos, accounting for production of over 40% of *Ae. aegypti* pupae [50].

### Comparison between Skeeter Buster and CIMSiM

CIMSiM and Skeeter Buster handle multiple containers of the same type in different ways. While CIMSiM models a single representative container, and multiplies the results according to the density of such containers per hectare, Skeeter Buster models each container individually. In order to compare these two approaches, we first set both CIMSiM and Skeeter Buster to have the equivalent of 100 containers of each of the above three types in an area of one hectare, with completely random mating of the mosquitoes within this area. For Skeeter Buster, this was equivalent to modeling a single “property” with a 1 ha yard in which 100 containers of each type are placed. We compare the outcome of this simulation to that of CIMSiM set up with the same three types of containers, each with a density of 100/ha. Both approaches model a similar 1-ha area. The primary difference is that Skeeter Buster models the dynamics in each of the 300 containers individually, whereas CIMSiM simulates the dynamics in groups of only 3 representative containers.

**Figure 7:**
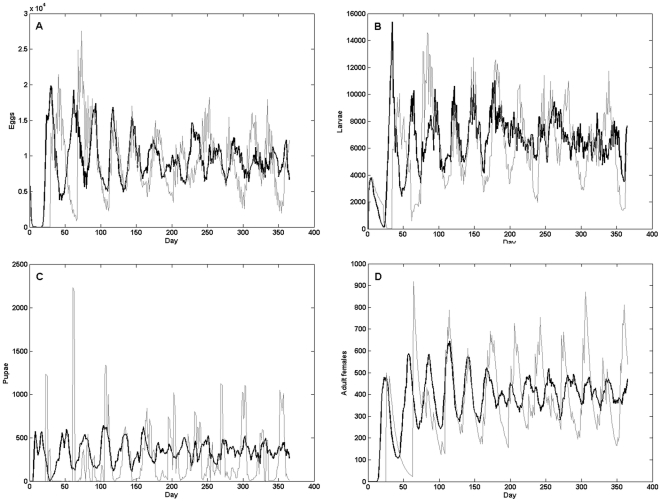
Time series comparisons between C++ CIMSiM and Skeeter Buster. Stage-specific time series from C++ CIMSiM (light gray line) and Skeeter Buster (black line). Containers are 1-gallon buckets, each simulation is set up with 100 containers in a single location. Weather data used were collected in Iquitos, Peru, 1978. A: Eggs; B: larvae; C: pupae; D: female adults.

We compared the stage-specific densities of eggs, larvae, pupae and adult females within the 1-ha area from single runs of both Skeeter Buster and CIMSiM (Figure 7). For all developmental stages of *Ae. aegypti*, a common characteristic of the output from Skeeter Buster is that the temporal variation in density is reduced compared to CIMSiM. Although it may appear paradoxical to observe less variation in a stochastic model, this result can be explained by two major differences between these two models. First, because of the stochasticity incorporated in Skeeter Buster, the demographic dynamics in each container are independent and not synchronized, which reduces the variability when the total density across all 300 containers is considered. Second, in Skeeter Buster, individuals within a given larval cohort do not necessarily all pupate on the same day, and pupation can be spread across several days. The same effect applies for larvae maturation and pupae maturation. As a result, the ‘cohort effect’ is quickly lost in the simulation, reducing the temporal variation in densities.

The average stage-specific densities, taken over the entire year, in Skeeter Buster are similar to those obtained from CIMSiM. Minor differences in average densities can be explained by the different daily mortality rates used in Skeeter Buster, or by minor changes in the oviposition procedures (see Text S4). These changes also affect the periodicity of these time series, with the interval between peaks of female adult densities appearing to be slightly shorter in Skeeter Buster (see Figure 7D, and Text S3, Figure S5 and Figure S6 for a more detailed analysis of time series periodicity).

### Impact of spatial structure and heterogeneity

We incorporate spatial structure in Skeeter Buster by considering simulations using the same 300 containers (100 of each type) as before, but now distributed among 100 individual properties. Properties are laid out on a 10×10 grid, and migration between individual properties can occur (see Methods). To explore the impact of habitat heterogeneity, we consider two container distributions. First, a homogeneous container distribution in which each property has exactly 3 containers, 1 of each type; in other words, all properties have an identical container distribution. Second, a heterogeneous container distribution, in which all 300 containers are randomly assigned to one of the 100 properties. In this case, the overall number of containers remains the same as in the homogenous case, but individual properties can have different types and numbers of containers.

**Figure 8:**
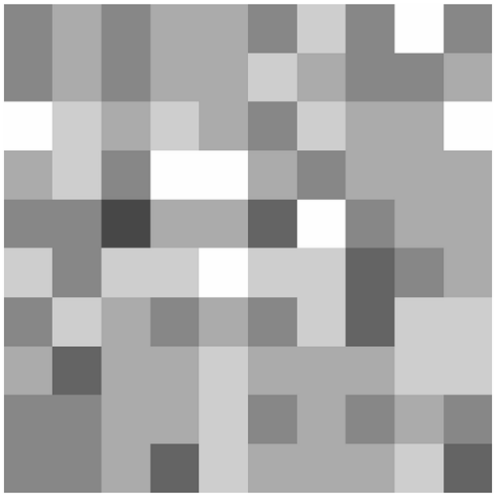
Spatial representation of the population simulated by Skeeter Buster. Spatial representation of the pupal composition of the population after a 1-year simulation using 100 properties each containing 3 containers (one of each type described in the text). Each square represents an individual property. The grayscale represents the number of pupae found at this property on day 365. Properties with no pupae are colored in white, whereas the presence of pupae is denoted in gray, with darker shades representing higher numbers of pupae.

**Figure 9:**
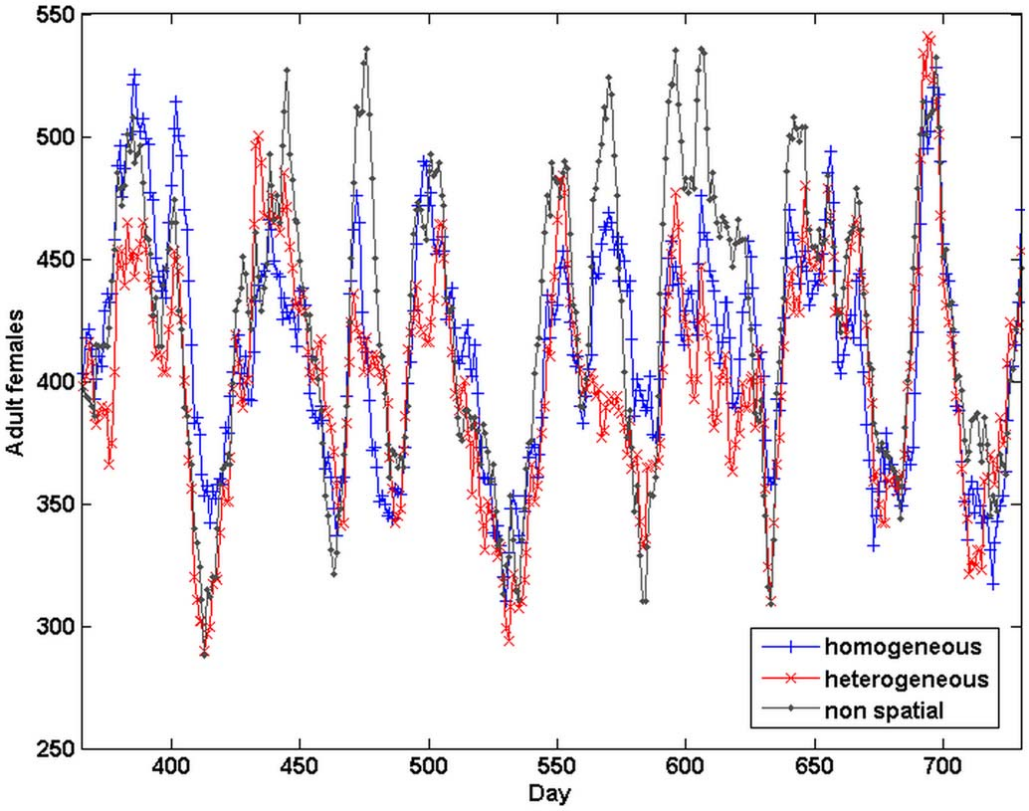
Effects of habitat heterogeneity on female adult densities. Time series of the total number of female adults in the population from three different Skeeter Buster simulations. All three setups use 100 containers of each type (see text for description). Non-spatial (gray line) is a single property containing all 300 containers. Homogeneous distribution (blue line) means 100 properties each containing exactly 3 containers (one of each type). Heterogeneous distribution (red line) means 100 properties with the 300 containers randomly distributed amongst them. In all cases, the model is initialized with egg cohorts only. The results presented are for year 2 of the simulation. Respective mean number of females +/−SD are: homogeneous: 413.8+/−46.5 ; heterogeneous: 401.9+/−47.3 ; 425.4+/−57.4.

We present a snapshot of the spatial variation in the density of the population, as the number of pupae per property, at the end of a 1-yr simulation with the homogeneous container distribution, on Figure 8. Because of the effects of both stochasticity in local dynamics and dispersal, there is clear spatial heterogeneity among population densities between individual properties, even when their container composition is the same. We compare the time series of female adult density in the whole population for both types of habitat heterogeneity described above, as well as for the non-spatial case described in the previous section (Figure 9). Both average densities and temporal variances are comparable in all three cases, and therefore do not appear to be affected by habitat heterogeneity.

**Figure 10:**
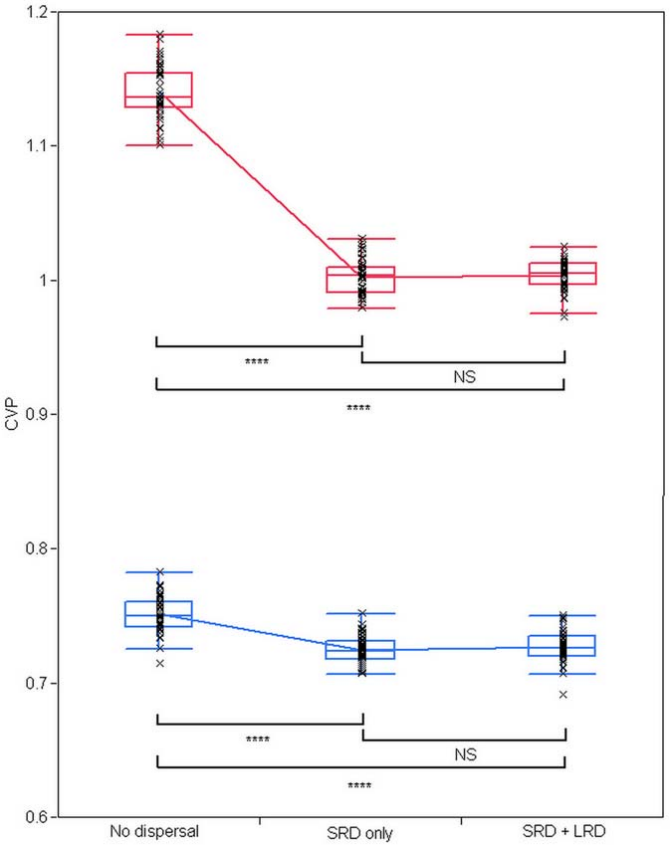
Effects of habitat heterogeneity and adult dispersal on density heterogeneity between properties. Effects of habitat heterogeneity and adult dispersal on the spatial coefficient of variation of the number of pupae among properties (CV_p_). Two container distributions are considered (see text for details): homogeneous (blue line and boxes), or heterogeneous (red line and boxes). Three adult dispersal patterns are considered: (i) no dispersal; (ii) short range dispersal (SRD) only, and (iii) both short range dispersal and long range dispersal (LRD). For both (ii) and (iii), short range dispersal occurs with daily probability of 0.3 per adult per day. For (iii), long range dispersal occurs with daily probability of 0.02 per adult per day. For each combination (container distribution×dispersal pattern), 50 simulations are run. For each simulation, the plotted value of CV_p_ is calculated as the average of the daily CV_p_ value for the last 100 days of the simulation. The result from each simulation is represented by a ‘x’ symbol. Boxes show, for each combination, the 25% and 75% quantiles. The middle line in the box represents the median and the whiskers encompass the data points that fall within 1.5 interquartile range in each direction. The lines between boxes connect the means. Within each container setup, pairwise mean comparisons are tested by Student's t-test (NS: p>0.05 ; ****: p<0.0001).

Habitat heterogeneity however has a strong effect on the level of spatial variation (between properties) in the population. We quantify this variation by measuring the coefficient of variation in the number of pupae among individual properties at a given time (denoted as CV_p_). We measured CV_p_ (Figure 10) in the two above-defined setups (homogeneous or heterogeneous), and under three different assumptions about adult dispersal between properties : (1) both short range and long range dispersal are allowed, with daily probabilities of 0.3 and 0.02, respectively; (2) only short range dispersal is allowed, or: (3) no dispersal at all. The results of analysis of variance for CV_p_ are also summarized (Table 1).

**Table 1:** Analysis of variance in CV_p_ values testing for the effects of habitat heterogeneity dispersal pattern.

Source	df	Sum of squares	F	p
Habitat heterogeneity	1	7.413	41713.1	<0.0001
Dispersal	2	0.446	1254.16	<0.0001
Habitat het.×Dispersal	2	0.210	591.31	<0.0001
Error	294	0.052		
Total	299	8.121		

CV_p_ = coefficient of variation in the number of pupae among individual properties. Habitat heterogeneity can be homogeneous or heterogeneous. Types of dispersal can be: no dispersal, short range dispersal only, or both short and long range dispersal. df = degrees of freedom, F = F-statistic.

These results show a clear effect of the spatial distribution of containers on CV_p_. As expected, the values of CV_p_ are significantly higher when the container distribution is heterogeneous. Dispersal also has a significant effect. For both container distributions, CV_p_ is significantly higher when no dispersal occurs. On the other hand, the values of CV_p_ when short and long range dispersal occur do not differ from the case when only short range dispersal is allowed, suggesting that long range dispersal does not affect spatial variance among properties within the specified level of heterogeneity. Similarly, there is a significant effect of the interaction between container distribution and dispersal pattern. The effects of dispersal on CV_p_ are more pronounced when the container setup is heterogeneous.

### Effects of adult movement and habitat heterogeneity on genetic structure of the population

Finally, we describe how the genetic structure of the population is affected by spatial factors such as the distribution of containers (homogeneous or heterogeneous) and adult dispersal. We follow the dynamics of a single locus with two alleles that do not differentially impact fitness (*i.e.* two neutral alleles). Both alleles are initially introduced into the population in egg cohorts homozygous for one of the two alleles, each at a frequency of 0.5. Simulations are set up with 400 properties (20×20 grid), with the same three container types as above, and run for 5 years. We arbitrarily define 25 subpopulations that consist of non-overlapping 4×4 squares within the 20×20 grid. (Here, we use 400 properties instead of 100 to allow us to partition our grid into a larger number of subpopulations, facilitating the spatial analysis that follows.) Short range dispersal is set to its default value (0.3 daily dispersal probability), and we examine the effects of varied amounts of long range adult and container movement on the genetic structure of the population.

**Figure 11:**
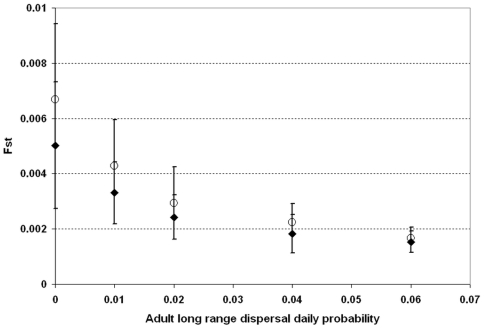
Effects of adult dispersal on the genetic structure of the population. Final F_ST_ values after a 5-year simulation of a 20×20 grid of properties subdivided in 4×4 squares. Calculation is based on one neutral marker, and two alleles introduced at equal frequency at every property in the population. Solid diamonds: homogeneous container distribution; Open circles: heterogeneous container distribution (mean+/−SD). LRD = long range dispersal. 20 simulations are run for each container distribution×dispersal assumption combination.

We calculated the global F_ST_ values based on this neutral locus at the end of the simulations (Figure 11). F_ST_ values, representing the level of genetic differentiation within the overall population (between subpopulations), are higher in the case of a heterogeneous distribution of containers, but decrease quickly when the daily probability of long range dispersal increases.

**Figure 12:**
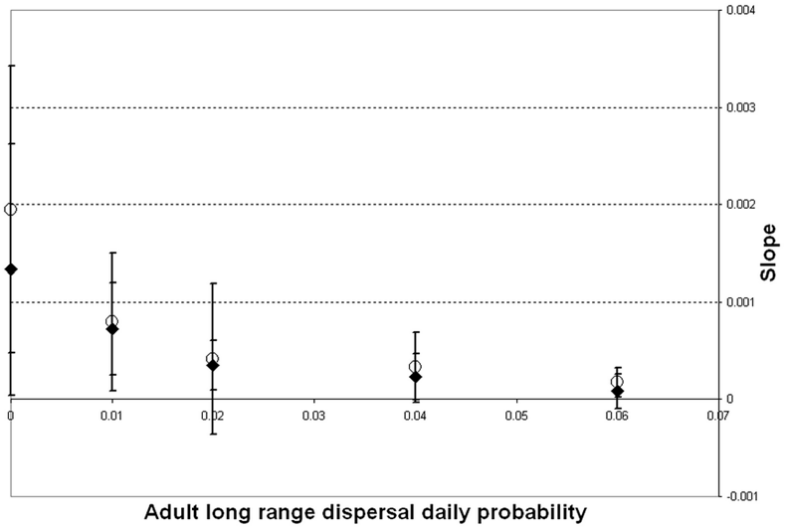
Effects of adult dispersal on the existence of isolation by distance in the population. Slope of the linear regression of F_ST_/(1−F_ST_) against the geographic distance between pair of subpopulations at the end of the simulation (see Fig. 10 for a description of the simulations), as a function of the daily probability of long range dispersal. Solid diamonds: homogeneous container distribution ; Open circles: heterogeneous container distribution.

We also calculate pairwise F_ST_ values between all 25 subpopulations. We can test the existence of isolation by distance in our simulated population by examining the correlation between the genetic distance between two subpopulations (given by the pairwise F_ST_ value) and their geographic distance. More specifically, following the method described in [52], we regress the values of F_ST_/(1−F_ST_) for pairs of subpopulations against the logarithms of their geographic distances. Isolation by distance is characterized by a significant correlation between these two distances. A stronger isolation by distance is associated with a higher slope of the regression line. We measured the values of this slope for different assumptions concerning habitat heterogeneity and adult movement (Figure 12). For both types of container distribution, long range dispersal, even at daily probabilities as low as 0.02, prevents the occurrence of isolation by distance at the scale of the simulation considered here (20×20 properties).

**Figure 13:**
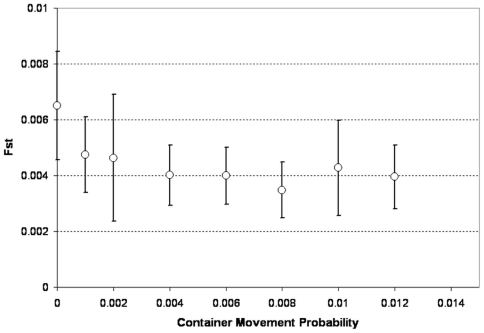
Effects of container movement probability on the genetic structure of the population. Final F_ST_ values after a 5-year run (see Fig. 10 for a description of the simulation). Symbols are the average value across 10 repetitions, error bars are SD. Container movement probability is the daily probability for each container of being moved to another property. Because container movement would rapidly lead an initially homogeneous container distribution to become heterogeneous, here we only present simulations that employ the heterogeneous container distribution.

Finally, we also examine the impact of container displacement (and the associated movement of egg cohorts) between properties. We measured the impact of this movement on final F_ST_ values for a neutral allele, assuming that there is no long range dispersal (Figure 13). Only the plastic buckets (1-gallon and 5-gallon) are moved since larger containers are not typically moved among households. It appears that moving containers across the city can have an impact on the population structure even when these events are rare, although increasing this probability does not seem to impact F_ST_ values as much as adult dispersal.

## Discussion

The results from Skeeter Buster presented in this paper using simplified container and property setups highlight the impact of spatial structure and heterogeneity on the population dynamics of *Ae. aegypti*. First, the simulated population dynamics differ markedly between CIMSiM and Skeeter Buster when a large number of identical containers within one property are considered in Skeeter Buster. Because each of these containers is simulated individually in the stochastic Skeeter Buster, the overall population dynamics is an average over a large number of containers whose individual dynamics are typically not synchronized. Additionally, containers in different properties are associated with a different local population. Identical containers in Skeeter Buster can therefore exhibit very different dynamics from one another. As a consequence, the variability in densities of *Ae. aegypti* at the level of the population is greatly reduced (see Fig. 7).

Beyond the effect of simulating individual containers, the explicit simulation of individual properties in Skeeter Buster does not seem to affect the overall population dynamics, at least in the settings investigated here (see Fig. 9). However, this inclusion of multiple properties allows a quantitative description of spatial heterogeneity among properties in terms of *Ae. aegypti* densities and age composition that could not be modeled by CIMSiM. We show here that the level of heterogeneity among properties in *Ae. aegypti* population density can be high even when a homogeneous container distribution is considered. Future studies based on Skeeter Buster will reveal if and how much this heterogeneity is predicted to affect both dengue transmission dynamics and the impact of vector control strategies. Because there is evidence that heterogeneity among properties in densities of female adults could be important for both [49],[53], we conclude that it is an important feature to include in our modeling tool.

Among the possible strategies for decreasing dengue incidence, approaches using genetic tools to control the mosquito population appear to be promising, but their applicability in field situations is still under evaluation. Skeeter Buster was designed to aid this evaluation, and simulate the efficiency and practicality of these approaches in order to guide the development of genetic control programs. We therefore incorporated explicit genetics in the model, and describe here the basic population genetic structure predicted by this model. While long range dispersal does not seem to affect the spatial variance in densities, Figs. 11 and 12 show that long range dispersal can significantly affect the genetic structure of the population. Even relatively rare long range dispersal events (daily probability lower than 2%) are associated with lower F_ST_ values in the population and dramatically reduce the observed instances of isolation by distance among subdivisions of our modeled population. The transfer of containers between properties in the grid can also impact the genetic structure, although its impact does not seem to be as important as that of adult long range dispersal (Figure 13). The existence of strong genetic spatial structure in the population is important to the potential fate of an allele introduced into specific locations within a population. Strong genetic structure could impede or slow the spread of a novel allele to distant parts of the population. For this reason, the ability of Skeeter Buster to model this genetic structure is an important addition for predicting the outcome of genetic control strategies in *Ae. aegypti* populations.

The spatial scale examined in Skeeter Buster is at the level of individual properties, that is, in the case of Iquitos, distances of an order of magnitude of hundreds of meters. Field studies of genetic structure at this level are rare. F_ST_ values reported from small-scale clusters (kilometers) in within-city studies [54]–[57] are variable but consistent with the highest values observed in the simulations presented in this paper. This would suggest a limited amount of adult dispersal between these geographically close sites, without excluding the possibility of gene flow maintained by displacement of immatures through human activities and transportation. More generally, these results emphasize the need to characterize the dispersal patterns of *Ae. aegypti* in natural populations. While adults are generally considered to migrate only short distances (modeled by our short range dispersal) [45], dispersal to longer distances has been observed [46], but how often such long range dispersal events occur is unknown.

Overall, the results presented here are consistent with our assertion that Skeeter Buster provides a realistic description of *Ae. aegypti* population dynamics and will be a valuable tool in the development of city-wide genetic strategies for prevention of dengue and control of its major mosquito vector. Ultimately, this entomological simulation will be a component of a framework from which dengue transmission can be modeled, and control measures can be evaluated. However, two important requirements have to be fulfilled before these further steps can be carried out. First, the outcome of the model must be validated with population data from an actual field site: this will rely on a more elaborate property setup and container distribution than the examples presented here. Skeeter Buster allows for detail at the individual container level, and therefore enables a specific *Ae. aegypti* population in a particular location to be modeled. But, to achieve such a location-specific level of accuracy, Skeeter Buster requires intensive field work to obtain a description of the container distribution and relative productivity in this particular location. In a subsequent paper, we will illustrate this location-specific simulation capacity with a case study of the city of Iquitos, Peru. Second, since this model relies on a very high number of procedures and parameters, all of which are associated with some level of uncertainty, it is crucial to carry a broad-scale uncertainty and sensitivity analysis of this model. This analysis will ensure that the results of the simulations are robust enough within the range of the existing uncertainties on parameter values, or, if not, the analysis will highlight the traits predicted to account for the highest percentage of uncertainty in predicted population dynamics and genetics, providing guidelines for the most needed additional field or laboratory studies.

## Supporting Information

Dataset S1 Spreadsheet that references and summarizes relevant publications on *Ae. aegypti*.
(0.22 MB XLS)

Figure S1 Discrepancies between uncorrected C++ CIMSiM and original CIMSiM. Number of larvae for C++ CIMSiM (green) without the corrections detailed in the text and in the absence of manipulations, and for the original CIMSiM (red). Weather data was collected for Iquitos, Peru 1978. Containers used were 1 gallon buckets.
(0.03 MB TIF)

Figure S2 Details of the discrepancies between uncorrected C++ CIMSiM and original CIMSiM and associated cohort manipulations. Differences in the number of eggs (red), larvae (green) and pupae (blue) between uncorrected C++ CIMSiM (in the absence of the cohort manipulations discussed in the text) and the original CIMSiM. Weather data was collected for Iquitos, Peru 1978. Containers used were 1 gallon buckets. Arrows mark days 158 and 232 at which malfunctions occur in the original CIMSiM.
(0.08 MB TIF)

Figure S3 Cumulative proportion of larvae reaching physiological development based on the current physiological status of the cohort. For values of CDt (cumulative physiological development) between 0.89 and 1.17, a certain proportion of larvae within the cohort can become developed. In Skeeter Buster, the actual number of larvae becoming developed is drawn from a binomial distribution (see the calculation of the probability associated to this distribution in the text). Note that 50% of larvae are expected to become mature before the cumulative physiological development reaches 1.0, and 50% after.
(0.03 MB TIF)

Figure S4 Pupation windows as a function of physiological development status and temperature. Pupation windows define the required minimal larval weight for pupation. Lines correspond, from top to bottom, to temperatures of 15, 20, 25, 30 and 35°C. Symbols represent hypothetical larval trajectories from simulation using weather data from Iquitos, Peru, under different nutritional conditions (dark blue: high food; red: medium; light blue: low food) and show the progress of these cohorts in terms of weight gain and cumulative physiological development (CDt). Pupation of these larvae occurs when their trajectory crosses the pupation window.
(0.01 MB EPS)

Figure S5 Periodogram of female adult density from C++ CIMSiM and Skeeter Buster. This periodogram is based on a discrete Fourier transformation of the time series presented in the main text (Fig. 6D). The dominant period of the cycles is approximately two days shorter in Skeeter Buster, resulting in approximately 13 density peaks a year, compared to the 12 peaks predicted by C++ CIMSiM.
(0.02 MB TIF)

Figure S6 Periodograms of female adult densities for various setups of Skeeter Buster. These periodograms are based on discrete Fourier transformation of time series from the model. All simulations are run using 1-gallon buckets and weather data from Iquitos, Peru, 1978–1980. Simulations are run for three years. To avoid initial cohort effects, only the last two years of each time series is analyzed. Moreover, the population is initialized with cohorts from all life stages, in proportions defined by a run of Skeeter Buster with non-limiting food. The left column represents simulations with no spatial structure, and 100 containers within the same location. The right column represents simulations with spatial structure, and 100 properties, each containing one single container. In the latter case, only short range dispersal is allowed (there is no long range dispersal). Rows correspond to different food conditions, modeled as daily food gain per container: top row, low food amounts (0.8 mg/day) ; middle row, medium (default) food amount (1.8 mg/day) ; bottom row, high food amount (3.0 mg/day). Note that the y-axes have different scales between panels.
(0.07 MB TIF)

Text S1 Verification of C++ CIMSiM against the original CIMSiM.
(0.03 MB DOC)

Text S2 Details of CIMSiM elements used in Skeeter Buster, together with modifications adopted.
(0.13 MB DOC)

Text S3 Analyses of periodicities in the time series.
(0.02 MB DOC)

Text S4 List of differences in biological procedures between CIMSiM and Skeeter Buster.
(0.05 MB DOC)
