# Supplementary figures and images for: Skeeter Buster: A Stochastic, Spatially Explicit Modeling Tool for Studying Aedes aegypti Population Replacement and Population Suppression Strategies
Source: PLoS Negl Trop Dis. 2009 Sep 1;3(9):e508. doi: 10.1371/journal.pntd.0000508 (PMC2728493; doi:10.1371/journal.pntd.0000508)

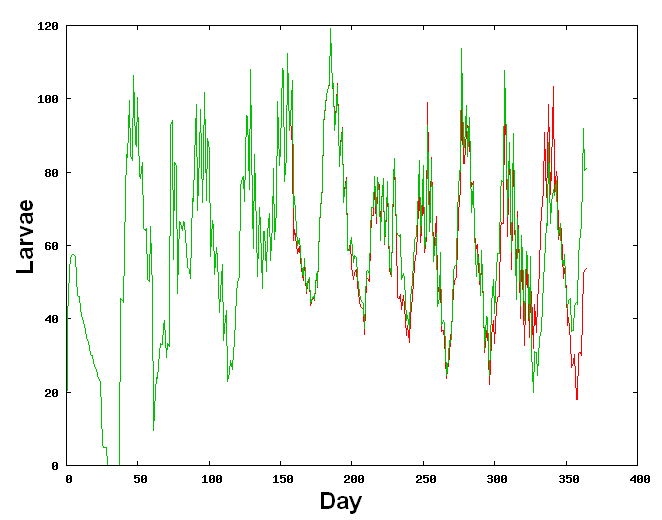

Supplement: Figure S1 — Discrepancies between uncorrected C++ CIMSiM and original CIMSiM. Number of larvae for C++ CIMSiM (green) without the corrections detailed in the text and in the absence of manipulations, and for the original CIMSiM (red). Weather data was collected for Iquitos, Peru 1978. Containers used were 1 gallon buckets. (0.03 MB TIF) [file pntd.0000508.s002.tif]

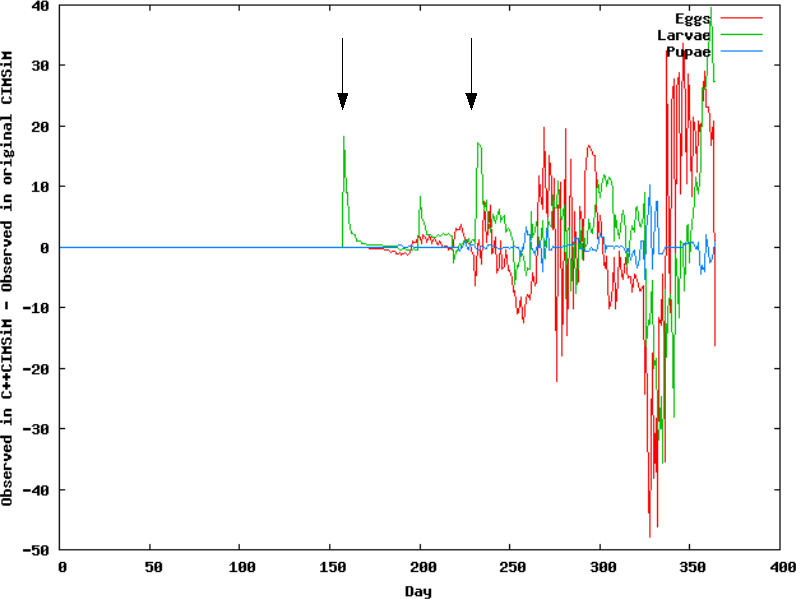

Supplement: Figure S2 — Details of the discrepancies between uncorrected C++ CIMSiM and original CIMSiM and associated cohort manipulations. Differences in the number of eggs (red), larvae (green) and pupae (blue) between uncorrected C++ CIMSiM (in the absence of the cohort manipulations discussed in the text) and the original CIMSiM. Weather data was collected for Iquitos, Peru 1978. Containers used were 1 gallon buckets. Arrows mark days 158 and 232 at which malfunctions occur in the original CIMSiM. (0.08 MB TIF) [file pntd.0000508.s003.tif]

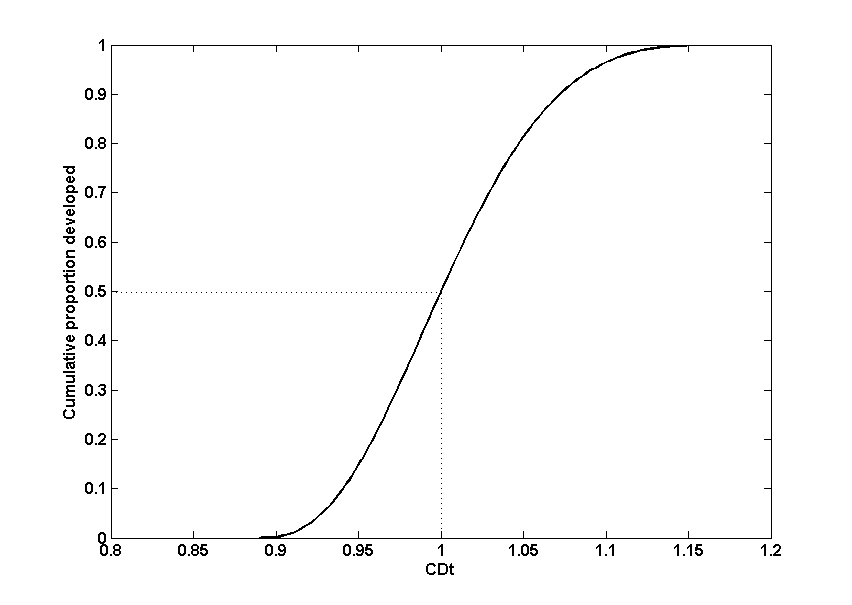

Supplement: Figure S3 — Cumulative proportion of larvae reaching physiological development based on the current physiological status of the cohort. For values of CDt (cumulative physiological development) between 0.89 and 1.17, a certain proportion of larvae within the cohort can become developed. In Skeeter Buster, the actual number of larvae becoming developed is drawn from a binomial distribution (see the calculation of the probability associated to this distribution in the text). Note that 50% of larvae are expected to become mature before the cumulative physiological development reaches 1.0, and 50% after. (0.03 MB TIF) [file pntd.0000508.s004.tif]

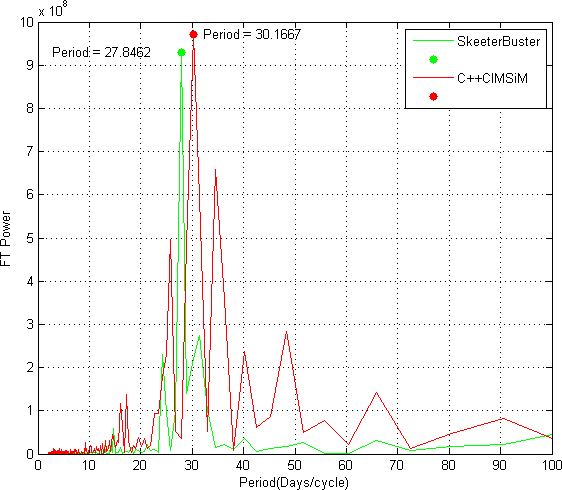

Supplement: Figure S5 — Periodogram of female adult density from C++ CIMSiM and Skeeter Buster. This periodogram is based on a discrete Fourier transformation of the time series presented in the main text (Fig. 6D). The dominant period of the cycles is approximately two days shorter in Skeeter Buster, resulting in approximately 13 density peaks a year, compared to the 12 peaks predicted by C++ CIMSiM. (0.02 MB TIF) [file pntd.0000508.s006.tif]

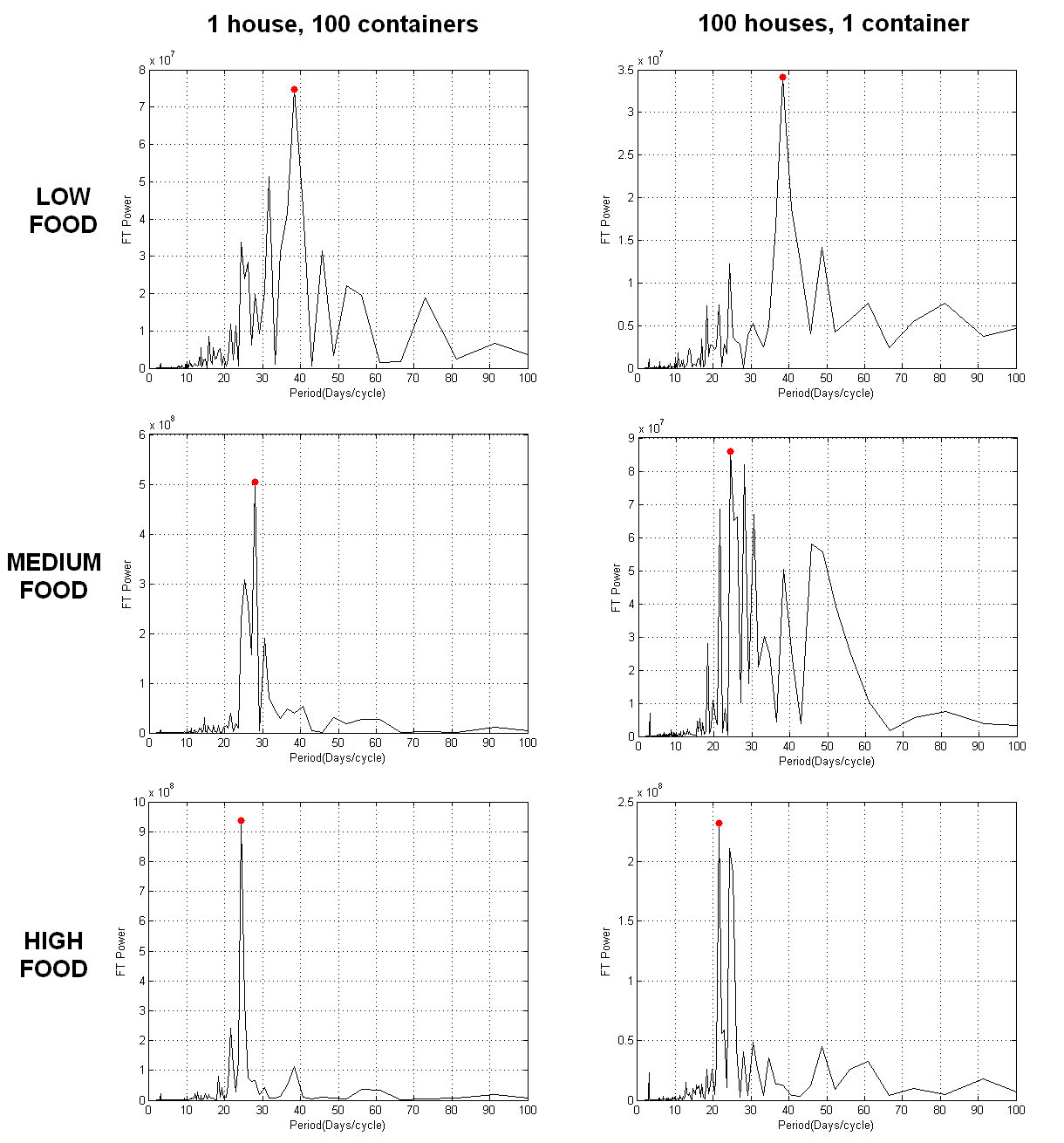

Supplement: Figure S6 — Periodograms of female adult densities for various setups of Skeeter Buster. These periodograms are based on discrete Fourier transformation of time series from the model. All simulations are run using 1-gallon buckets and weather data from Iquitos, Peru, 1978–1980. Simulations are run for three years. To avoid initial cohort effects, only the last two years of each time series is analyzed. Moreover, the population is initialized with cohorts from all life stages, in proportions defined by a run of Skeeter Buster with non-limiting food. The left column represents simulations with no spatial structure, and 100 containers within the same location. The right column represents simulations with spatial structure, and 100 properties, each containing one single container. In the latter case, only short range dispersal is allowed (there is no long range dispersal). Rows correspond to different food conditions, modeled as daily food gain per container: top row, low food amounts (0.8 mg/day) ; middle row, medium (default) food amount (1.8 mg/day) ; bottom row, high food amount (3.0 mg/day). Note that the y-axes have different scales between panels. (0.07 MB TIF) [file pntd.0000508.s007.tif]
