# Supplementary material for: Skeeter Buster: A Stochastic, Spatially Explicit Modeling Tool for Studying Aedes aegypti Population Replacement and Population Suppression Strategies
Source: PLoS Negl Trop Dis. 2009 Sep 1;3(9):e508. doi: 10.1371/journal.pntd.0000508 (PMC2728493; doi:10.1371/journal.pntd.0000508)
Supplement: Text S1 — Verification of C++ CIMSiM against the original CIMSiM. (0.03 MB DOC) [file pntd.0000508.s008.doc]

**Text S1:**

**Verification of C++ CIMSiM against the original CIMSiM**

We chose to rewrite a clone (named C++ CIMSiM) of the existing CIMSiM model [1] as a starting point for the development of Skeeter Buster. This rewriting allowed us to identify a number of inconsistencies between the original CIMSiM model as coded and the algorithms presented in the corresponding paper [1]. We list these here, along with the corrections made when these were imported into C++ CIMSiM and Skeeter Buster.

- In CIMSiM, the weight of dead pupae for the next day is erroneously inflated by multiplying the overall survival of pupae with survival at eclosion even for pupal cohorts not emerging. This error is not present in C++ CIMSiM or Skeeter Buster.
- In CIMSiM, the weight of dead pupae that died during eclosion is not included into the cadaver weight for the next day. In C++ CIMSiM and Skeeter Buster, it is included.
- In CIMSiM, 26 ºC is converted to Kelvin as 299 K. In C++ CIMSiM and Skeeter Buster, 26 ºC is converted to the more accurate 299.15 K. Although this seems like a small difference, due to the sensitivity of larval maturation and growth rates on temperature, it can cause the larvae to pupate a day earlier in C++ CIMSiM and Skeeter Buster than in CIMSiM.
- In CIMSiM, prefasting lipid reserve can be negative at very small larval weights according to Equation 9 in [1]. In C++ CIMSiM and Skeeter Buster, this is corrected by setting it to zero in such instances.
- In CIMSiM, the developmental rate at the developmental threshold of 13.4ºC is inaccurately fixed at 0.00146 hr^-1^. In C++ CIMSiM and Skeeter Buster, it is accurately calculated as 0.0347 hr^-1^.
- In CIMSiM, half as many eggs are laid as should have been when no females emerge for five days and outdoor containers are used (fecundity calculation is different in outdoors and indoor containers). The same happens on any day when there are no female nulliparous adults. This does not happen in C++ CIMSiM or Skeeter Buster.

Verification of a version of C++ CIMSiM (in which the above six imprecisions were deliberately left uncorrected) to the original CIMSiM revealed persisting differences between the output of these two models. Only deliberate manipulation of specific cohorts on specific dates in the source code of C++ CIMSiM allowed us to get rid of these mismatches. Here we present the details and the rationale for these manipulations.

We compare the number of larvae in C++ CIMSiM in the absence of these manipulations to the number of larvae based on the original CIMSiM executable (Figure S1). The number of larvae matches exactly until day 158 with the specific parameters used (Iquitos, Peru 1978 weather data, 1 gallon buckets). At day 158, there are 18.3 more larvae according to C++ CIMSiM relative to the output of the original program (Figure S2), while all other life stages are identical. We argue that this difference is due to the 'disappearance' of a cohort of freshly hatched larvae in the original program. This is based on the evidence that this difference is identical to the size of a specific egg cohort that hatches on this day in C++ CIMSiM, according to the algorithms (see main text Figure 2) that should also apply to the original program. Moreover, when we artificially remove this specific egg cohort in C++ CIMSiM, numbers of larvae are identical between the two programs up until day 232 when a similar discrepancy occurs. When we perform the same manipulation on day 232, we get perfect correspondence between the outputs of the two programs over the entire one-year simulation period (see main text Figure 5).

We considered alternative hypotheses to explain this presence of surplus larvae in C++ CIMSiM relative to the original program. This excess of larvae could be due to an increase of eggs hatching or a decrease of larvae pupating on that specific day in C++ CIMSiM. However, numbers of both eggs and pupae are identical between C++ CIMSiM and the original CIMSiM for that specific day (Figure S2). It is also possible that larval mortality would be decreased in C++ CIMSiM on the specific day. One consequence of such a decrease would be a decrease in the amount of larval food in the containers. Since the amount of larval food in the containers is equal in C++ CIMSiM and the original program, we reject this hypothesis. Finally, the surplus larvae in C++ CIMSiM could be the result of an unknown error or a duplication. However, we can identify the unique egg cohort on the preceding day from which the excess larvae hatch, exactly following the algorithms in Figure 3. Unfortunately, the original CIMSiM output does not allow us to examine the development of single cohorts as C++ CIMSiM does. However, in light of the above indirect evidence, we are confident that the observed differences can only be explained by a malfunction of the original CIMSiM executable. (Potential causes could include a memory allocation error in the code or a bug in the compiler used to generate the executable.)

**Reference:**

1. Focks DA, Haile DG, Daniels E, Mount GA (1993) Dynamic life table model o*f Aedes aegyp*ti (Diptera: Culicidae) - Analysis of the literature and model development. J Med Entomol 30: 1003-1017.
