# Supplementary material for: Skeeter Buster: A Stochastic, Spatially Explicit Modeling Tool for Studying Aedes aegypti Population Replacement and Population Suppression Strategies
Source: PLoS Negl Trop Dis. 2009 Sep 1;3(9):e508. doi: 10.1371/journal.pntd.0000508 (PMC2728493; doi:10.1371/journal.pntd.0000508)
Supplement: Text S2 — Details of CIMSiM elements used in Skeeter Buster, together with modifications adopted. (0.13 MB DOC) [file pntd.0000508.s009.doc]

**Text S2 :**

**Details of CIMSiM elements used in Skeeter Buster, together with modifications adopted**

**S2.1. The amount and temperature of water in the containers**

Water temperature is a critical determinant of immature mosquito developmental and survival rates. Skeeter Buster, following CIMSiM, assumes that water temperature fluctuations depend on air temperatures, solar exposure and container characteristics. The dependence of water temperature and evaporative loss on these factors was estimated by D.A. Focks in an unpublished study undertaken in Gainesville, FL, USA. In this study, water levels and temperatures of 12 containers were monitored for 76 days, and regressed against meteorological data from an adjacent weather station, giving the following relationships:

$$\begin{matrix} WaterTemp_{\text{max}}=15.03+0.27AirTemp_{\text{min}}+0.01AirTemp_{\text{max}}^{2}+7.69SunExp^{2} \\ WaterTemp_{\text{min}}=5.02-1.36SunExp+0.81AirTemp_{\text{min}}+0.001AirTemp_{\text{max}}^{2} \end{matrix}$$

*SunExp* represents the solar exposure of the container, expressed as a proportion between 0 = complete shadow and 1 = maximal sunlight. *AirTemp*_min_ and *AirTemp*_max_ are daily minimal and maximal temperatures, respectively. For containers whose volume is larger than 5 litres, moving averages of the daily values of both *WaterTemp*_min_ and *WaterTemp*_max_ are used to mimic the attenuated temperature fluctuations due to thermal inertia. The length of this moving average is increasing for increasing volume of the containers (4 days for >500l, 3 days for 100-500 l and 2 days for 50-100l). *WaterTemp*_min_ and *WaterTemp*_max_ are used in calculating survival probabilities as a function of temperature extremes; their average is used in thermal development calculations.

Daily evaporative loss (in cm) for uncovered containers is given by:

$$\begin{matrix} EvaporativeLoss=0.93+0.28SunExp-0.01RH \\ \end{matrix}$$

where *RH* is the atmospheric relative humidity (in percentage). Evaporative loss in covered containers is reduced compared to this by a user-specified amount. In the case of manual drawdown, total loss includes both evaporative loss and drawdown. Daily water gains in rain-filled containers are the product of rainfall and the watershed ratio of the container. Watershed ratio reflects the surface actually receiving rainfall relative to the actual area of the container – containers associated with an active rain collection system have a watershed ratio >1, while containers like bottles with a narrow opening have a ratio <1. Manually-filled containers are assumed to retain a constant water level.

**S2.2. The calculation of temperature-dependent developmental rates**

Skeeter Buster, following CIMSiM, determines the developmental rates of mosquitoes based on an existing enzyme kinetics model [1]. This model assumes that the rate of development is determined by a single rate-controlling enzyme which is reversibly denaturated at high and low temperatures. Skeeter Buster (and CIMSiM) uses a simplified version of this model [2] which assumes inactivation only at high temperatures. The developmental rate is calculated by:

$r(T_{t})=\frac{\rho_{(25^{^{\circ}}\text{C})}\frac{T_{t}}{298}e^{\frac{\Delta H_{\text{A}}^{\neq}}{R}(\frac{1}{298}-\frac{1}{T_{t}})}}{1+e^{\frac{\Delta H_{\text{H}}}{R}(\frac{1}{T_{1/2\text{H}}}-\frac{1}{T_{t}})}}$,

where *r*(*T_t_*) is the developmental rate (hr^-1^) at temperature *T* (K) on day *t*, *T_t_* is the mean of the moving average of *WaterTemp*_min_ and *WaterTemp*_max_ for all immature stages, while it is the average of *AirTemp*_min_ and *AirTemp*_max_ for the gonotrophic development of adult female mosquitoes. Parameter estimates are obtained by comparison to observed data, using non-linear regression, and finding the best estimates through an iterative process with initial estimates taken from [3].The definitions of all other parameters, as well as their estimated values for each lifestage[4], are given in Table S1.

Table S1. Parameters of the temperature-dependent enzyme-kinetics developmental rate model

| ***Parameter*** | ***Definition*** | ***Eggs*** | ***Larvae*** | ***Pupae*** | ***Gonotrophic cycle*** |
| --- | --- | --- | --- | --- | --- |
| *ρ*_(25ºC)_ | Development rate per hour at 25ºC assuming no temperature inactivation of the critical enzyme (hr^-1^) | 0.01066 | 0.00873 | 0.01610 | 0.00898 |
| Δ*H*_A_^≠^ | Enthalpy of activation of the reaction catalyzed by the enzyme (cal/mol) | 10,798.18 | 26,018.51 | 14,931.94 | 15,725.23 |
| Δ*H*_H_ | Enthalpy change associated with high temperature inactivation of the enzyme (cal/mol) | 100,000 | 55,990.75 | -472,379 | 1,756,481.07 |
| *T*_1/2H_ | Temperature at which 50% of the enzyme is inactivated from high temperature | 14184.5 | 304.58 | 148.45 | 447.17 |

For a given cohort of age *n* at time *t*, the cumulative physiological development *CD_t_* is then given by:

$$CD_{t}=\sum_{\tau=t-n}^{t} r(T_{\tau})$$

Developmental rates accumulate up to the point when the physiological development is considered completed.

**S2.3. Variation in development time in Skeeter Buster**

In CIMSiM, larval and pupal cohorts reach complete physiological development, and are marked as “developed”, when their cumulative physiological development (*CD_t_*) exceeds 0.95. In Skeeter Buster, we relax this assumption, and allow some individuals to become developed with *CD_t_* < 0.95 while others have to reach a higher value to become developed.

More precisely, no larva becomes developed when *CD_t_* < 0.89. All larvae become developed if *CD_t_* > 1.17. If 0.89 < *CD_t_* < 1.17, a proportion of a given cohort becomes developed. The cumulative proportion *y_t_* of a cohort reaching development based on the value of *CD_t_* is given by the following function [5]:

$y_{t}=(1-z_{t})^{2.0126z_{t}^{2}}$,

where

$z_{t}=\frac{1.17-CD_{t}}{1.17-0.89}$.

The shape of this function is shown in Figure S3. The proportion of larvae becoming developed at time *t* is then given by (*y_t_ – y_t-1_*)/(1-*y_t-1_*) (assuming *y_t_ =*0 if *CD_t_* < 0.89 and *y_t_*= 1 if *CD_t_*> 1.17). In Skeeter Buster, the actual number of larvae becoming developed is calculated according to a binomial distribution with a probability equal to (*y_t_ – y_t-1_*)/(1-*y_t-1_*).

Calculations are identical for pupal development.

**S2.4. Larval weight change and fasting**

Larval weight change and the associated changes in the amount of food in the container are modeled in parallel according to the following equations [6]:

$$\frac{dW(t)}{dt}=af(T_{t})\left(W(t)^{b}\left(1-e^{-cF(t)}-d_{1}W(t)^{d_{2}}\right)\right)$$

$\frac{dF(t)}{dt}=-n(t)f(T_{t})W(t)^{b}\left(1-e^{-cF(t)}\right)$,

where *t* is time, *W*(*t*) is larval dry weight (mg), *F*(*t*) is the amount of food within the container (mg), *T_t_* is the temperature (K) at time *t* and *n*(*t*) is the number of larvae in the cohort. Parameter values and descriptions are detailed in Table S2

**Table S2. Parameter description and default values for larval weight and food amount calculations**.

| ***Parameter*** | ***Description*** | **Value** |
| --- | --- | --- |
| **a** | Conversion rate of consumed food to biomass | 0.3 |
| **b** | Exponent of increase of food exploitation rate with body weight | 0.8 |
| **c** | Change in food exploitation rate with food density (type II functional response) | 0.1 |
| **d_1_** | Metabolic weight loss of larvae when food is totally depleted | 0.016 |
| **d_2_** |  | 0.667 |

The original equations were calibrated at 26 ºC*.* The change in metabolic rate with temperature is described by the function *f*(*T_t_*), calculated as:

$f(T_{t})=f_{T}\frac{r(T_{t})-r(13.4 ^{^{\circ}}\text{C)}}{r(26 ^{^{\circ}}\text{C})-r(13.4 ^{^{\circ}}\text{C)}}$,

where *f_T_* is the value at 26 ºC (*f_T_* = 0.001). *r*(*T_t_*) is calculated as described above. 13.4 ºC is the lower developmental threshold [7] at which *f*(*T_t_*) is set to zero.

*W*(*t*) and *F*(*t*) are calculated each day for each cohort in each container using Euler’s method with a resolution of 8 steps per day.

The proportion of lipid for a larva of weight *W*(*t*) is modeled by the following equations:

$$L(t)=0.059\left(\ln W(t)+6.9\right)$$

$R(t)=L(t)-L_{min}$,

where *L*(*t*) is the total proportion of lipid, *L*_min_ (= 0.15) is the proportion of lipid involved in structural components and therefore not available as reserves, and *R*(*t*) is the proportion of available lipid reserves. The amount of lipid reserves determines mortality probabilities for fasting larvae as described in the main text (Fig. 4).

**S2.5. Pupation**

Larvae in a given cohort can pupate if they meet two required conditions: their cumulative physiological development has to exceed a minimal threshold, and their weight has to exceed a minimal threshold. The values of these thresholds are temperature-dependent, and are not independent: at a given temperature, the value of the weight threshold depends on the cumulative physiological development of the cohort.

In Skeeter Buster, as in CIMSiM, this interaction is represented by an L-shaped window (Figure S4) representing the minimal weight (*W*_min_) as a function of the cohort’s current cumulative physiological development (*CD_t_*). This L-shaped window is described by the following equations:

- the vertical line on the left represents the minimal cumulative physiological development for any temperature (*CD_t_* = 0.95).
- beyond this value, and up to the maximum possible value of *CD_t_* = 8.0 (above that value, larvae are removed from the population), the weight threshold for pupation decreases linearly with *CD_t_*. The precise line for a given temperature is defined by two points. First, at *CD_t_* = 0.95 the minimal weight *W*_min_ is a function of water temperature. In CIMSiM, this minimal weight is given by the following equation [5]:

$$W_{\min}=2.11-0.04T_{t}$$

(where *T_t_* is the temperature at time *t*). In Skeeter Buster, this equation is modified to introduce an additional level of stochasticity (see below). From this point, the required minimal weight decreases linearly with *CD_t_* (*i.e.* as larvae age physiologically) down to a minimal temperature-independent requirement of 0.1 mg at the maximal value of *CD_t_* = 8.0.

The progress of a given larval cohort can then be represented by its trajectory on this graph, reflecting the changes in larval weight and cumulative physiological development (see examples on Figure S4). Pupation occurs when this trajectory crosses the L-shaped window.

Skeeter Buster introduces additional detail in the calculation of this window. First, specific windows are calculated for male and female larvae, with a lower minimal weight for pupation of males.

Moreover, an additional level of stochasticity is introduced in the definition of this L-shaped window. Instead of defining a single window for a given temperature, 4 windows are defined, corresponding to cumulative pupation probabilities of 25%, 50%, 75% and 100% when the trajectories cross the successive windows. These 4 windows are defined by the calculation of the upper-left point, defining the value of *W*_min_ at *CD_t_ =* 0.95. These calculations are presented in Table S3. 50% female and male pupation weight thresholds are estimated from [8]. 25%, 75%, 100% pupation weight thresholds are estimated from [9].

**Table S3. Calculation of weight thresholds (*W*_min_) at *CD_t_* = 0.95, as a function of temperature (*T_t_***) and pupation probability in Skeeter Buster

|  | **Female larvae** | **Male larvae** |
| --- | --- | --- |
| 25% | *W*_min_ = 1.5411 - 0.0389 *T_t_* | *W*_min_ = 1.2275 - 0.0389 *T_t_* |
| 50% | *W*_min_ = 1.7994 - 0.0389 *T_t_* | *W*_min_ = 1.4494 - 0.0389 T_t_ |
| 75% | *W*_min_ = 2.3270 - 0.0389 *T_t_* | *W*_min_ = 1.9665 - 0.0389 *T_t_* |
| 100% | *W*_min_ = 2.8545 - 0.0389 *T_t_* | *W*_min_ = 2.4836 - 0.0389 *T_t_* |

**S2.6. Nominal daily survival rates**
CIMSIM and Skeeter Buster assume that survival at all stages is independent of age and density (except for indirect larval fasting effects). Nominal daily survival probabilities are given in Table S4.

**Table S4. Stage-specific nominal daily survival probabilities.**

|  | ***Eggs*** | ***Larvae*** | ***Pupae*** | ***Adults*** |
| --- | --- | --- | --- | --- |
| **Nominal daily survival** | 0.99 | 0.99 | 0.99 | 0.89 (females)  0.77 (males) |

These survival probabilities can be modified by the multiplication of factors reflecting additional sources of mortality, as described in subsections II.7 and II.8.

**S2.7. Temperature-dependent survival probabilities**

For all stages, daily survival probability is multiplied by an additional factor *s_T_* reflecting temperature effects on survival. The value of this factor depends on the daily extreme temperatures *T*_min_ and *T*_max_, and on 4 stage-specific threshold temperatures. We can write:

*s_T_ = s_T_*_min_ ** s_T_*_max_

where:

- if *T*_min_ < *T*_0_ then *s_T_*_min_ = 0.05
- if *T*_0_ < *T*_min_ < *T*_1_ then *s_T_*_min_ = 0.05 + 0.95(*T*_min_-*T*_0_)/(*T*_1_-*T*_0_) (in other words, survival increases linearly with *T*_min_ from 0.05 at *T*_0_ to 1.0 at *T*_1_)
- otherwise, *s_T_*_min_ = 1.0 (no effect of minimum temperature on survival)

and:

- if *T*_2_ < *T*_max_ <T_3_ then *s_T_*_max_ = 1 - 0.95(*T*_max_-*T*_2_)/(*T*_3_-*T*_2_) (in other words, survival decreases linearly with *T*_max_ from 1.0 at *T*_2_ to 0.05 at *T*_3_)
- if *T*_max_ > *T*_3_ then *s_T_*_max_ = 0.05
- otherwise, *s_T_*_max_ = 1.0 (no effect of maximum temperature on survival)

Values of the threshold temperatures for these four temperature-dependent survival factors are given in Table S5. Note that the temperatures used in the calculation of these factors are water temperatures for eggs, larvae and pupae, and air temperatures for adults.

**Table S5. Thresholds for temperature-dependent survival calculations (ºC)**

|  | ***Eggs*** | ***Larvae/Pupae*** | **Adults** |
| --- | --- | --- | --- |
| *T*_0_ | -14 | 5 | 0 |
| *T*_1_ | -6 | 10 | 4 |
| *T*_2_ | 30 | 39 | 40 |
| *T*_3_ | 47 | 44 | 50 |

**S2.8. Survival to desiccation**

Desiccation can be an additional source of mortality for eggs when a container is dried out. In that case, survival probability is multiplied by an additional factor *s_H_*. This factor depends on two quantities, the sun exposure *SunExp* of the container, and the atmospheric saturation deficit *SD* (in mBars) (reflecting both humidity and temperature).

For containers with *SunExp*>0.85, *s_H_* = 0.95.

For containers with *SunExp*<0.85:

- - if *SD* < 10, *s_H_* = 0.99
  - if 10 < *SD* < 30, *s_H_* = 0.99 – 0.04*(*SD*-10)/(30-10) (in other words, survival decreases linearly with *SD* from 0.99 at *SD*=10 to 0.95 at *SD*=30)
  - if *SD*>30, *s_H_* = 0.95

Desiccation also affects adult survival when *SD* > 10 mBars. A similar survival factor *s_H_* multiplies daily adult survival in this case, and is calculated as follows:

- - if 10 < *SD* < 30, *s_H_* = 1 – 0.4*(*SD*-10)/(30-10) (in other words, survival decreases linearly with *SD* from 1.0 at *SD*=10 to 0.6 at *SD*=30)
  - if *SD*>30, *s_H_* = 0.6

**References:**

1. Sharpe PJH, DeMichele DW (1977) Reaction kinetics of poikilotherm development. J Theor Biol 64: 649-670.

2. Schoolfield RM, Sharpe PJH, Magnuson CE (1981) Non-linear regression of biological temperature-dependent rate models based on absolute reaction-rate theory. J Theor Biol 88: 719-731.

3. McHugh CP, Olson JK (1982) The effect of temperature on the development, growth and survival of *Psorophora columbiae*. Mosq News 42: 608-613.

4. Focks DA, Haile DG, Daniels E, Mount GA (1993) Dynamic life table model of *Aedes aegypti* (Diptera: Culicidae) - Analysis of the literature and model development. J Med Entomol 30: 1003-1017.

5. Rueda LM, Patel KJ, Axtell RC, Stinner RE (1990) Temperature-dependent development and survival rates of *Culex quinquefasciatus*  and *Aedes aegypti* (Diptera: Culicidae). J Med Entomol 27: 892-898.

6. Gilpin ME, McClelland GAH (1979) Systems-analysis of the yellow fever mosquito *Aedes aegypti*. Forts Zool 25: 355-388.

7. Bar-Zeev M (1958) The effect of temperature on the growth rate and survival of the immature stages of *Aedes aegypti*. Bull Entomol Res 49: 157-163.

8. Barbosa P, Peters TM, Greenough NC (1972) Overcrowding of mosquito populations: Responses of larval *Aedes aegypti* to stress. Environ Entomol 1: 89-93.

9. Chambers GM, Klowden MJ (1990) Correlation of nutritional reserves with a critical weight for pupation in larval *Aedes aegypti* mosquitos. J Am Mosq Control Assoc 6: 394-399.
