# Supplementary material for: Skeeter Buster: A Stochastic, Spatially Explicit Modeling Tool for Studying Aedes aegypti Population Replacement and Population Suppression Strategies
Source: PLoS Negl Trop Dis. 2009 Sep 1;3(9):e508. doi: 10.1371/journal.pntd.0000508 (PMC2728493; doi:10.1371/journal.pntd.0000508)
Supplement: Text S3 — Analyses of periodicities in the time series. (0.02 MB DOC) [file pntd.0000508.s010.doc]

**Text S3:**

**Analyses of periodicities in the time series**

The periodicity of the stage-specific time series appears to differ between C++CIMSiM and Skeeter Buster (see Figure 6D, main text). In this section we present analyses of the periodicities of these time series for various initial setups of the models, by plotting periodograms obtained through a discrete Fourier transformation of these time series.

According to a periodogram of female adult density for both models (Figure S5), the dominant period of female adult density fluctuations is about 2 days shorter in Skeeter Buster than it is in C++CIMSiM (approx. 28 days vs. 30 days).

We investigate how the periodicity is related to nutritional conditions and intraspecific competition within containers. We analyzed the periodicity of female adult density (Figure S6, left column) for three different nutritional conditions (defined as daily food gain per container in the model). It appears that the period of female adult density fluctuations is linked to the food availability in the containers (period of 24, 28 and 38 days for high, medium, and low food availability, respectively), although the dominant period is not clearly identifiable when the nutritional conditions are harsh.

Finally, the periodicity of female adult density at an individual property is strongly reduced when spatial structure is included, except when food levels are high (Figure S6, right column). In the latter case, the level of variation in development time among properties is greatly reduced, and fluctuations at the population level reflect those at the level of individual properties.  
