# Supplementary material for: Skeeter Buster: A Stochastic, Spatially Explicit Modeling Tool for Studying Aedes aegypti Population Replacement and Population Suppression Strategies
Source: PLoS Negl Trop Dis. 2009 Sep 1;3(9):e508. doi: 10.1371/journal.pntd.0000508 (PMC2728493; doi:10.1371/journal.pntd.0000508)
Supplement: Text S4 — List of differences in biological procedures between CIMSiM and Skeeter Buster. (0.05 MB DOC) [file pntd.0000508.s011.doc]

**Text S4:**

**List of differences in biological procedures between CIMSiM and Skeeter Buster**

We recapitulate here modifications made to biological procedures present in CIMSiM when we incorporated them into Skeeter Buster. (All six corrections discussed in Text S1 are also made.)

1. In CIMSiM, the calculation of the fecundity of female adult cohorts (made at the time of their emergence) is based on the moving average weight of the last five emerged female adult cohorts. While this is understandable as a simple solution to avoid keeping track of the weights and fecundities of all female adult cohorts separately, it has several drawbacks. First of all, it averages the weights of small-sized and large-sized adult cohorts, treating them as if they all have the average weight. Weights of nulliparous female adult cohorts are also included in this average. Additionally, this moving average is not weighted by the number of female adult mosquitoes in the different cohorts. In Skeeter Buster, we keep track of the weights and fecundities of all female adults separately.

2. In CIMSiM, all larvae in the same larval cohort pupate at the same time when they exceed their pupation weight threshold for the corresponding physiological percentage (2.11 mg at 26ºC for 0.95 cumulative physiological development). Males and females have the same parameters and pupate at the same time. In Skeeter Buster, four separate pupation windows are calculated with different parameters for male and female larvae (and parameter values are different to those used in the original CIMSiM). The four separate pupation windows (see Text S2.5) specify the weight the larvae have to achieve in order for 25%, 50%, 75% or 100% of them to pupate. (female at 26ºC 0.95 phys. dev. 25%: 1.5411, 50%: 1.79935, 75%: 2.32698, 100%: 2.854526; male at 26ºC 0.95 phys. dev. 25%: 1.2275, 50%: 1.44935, 75%: 1.9664689, 100%: 1.9664689) 50% female and male pupation weight thresholds are estimated from Figure 2 of [1]. 25%, 75%, 100% pupation weight thresholds are estimated from Figure 1 of [2].

3. In CIMSiM, the fecundity of female adult cohorts is independent of the age of the female adult cohort. In Skeeter Buster, fecundity of a female adult cohort is decreasing with age above age 25 with a slope of -0.4366743 eggs/day, based on Figure 3B of [3].

4. In CIMSiM, all larvae mature at the same time, when they reach cumulative physiological development *CD_t_* = 0.95. In Skeeter Buster, some larvae mature earlier and some later, according to [4]. The first larvae start to mature when they reach cumulative physiological development *CD_t_* = 0.89. All of the larvae mature when they reach cumulative physiological development *CD_t_* = 1.17. In between, only a fraction of the larvae mature based on equation 2 in [4]. In this case, a new mature cohort is branched off the original larval cohort with identical characteristics but marked as mature. Note that this cohort is not processed again on the same day.

5. In CIMSiM, all pupae mature at the same time, when they reach cumulative physiological development *CD_t_* = 0.95. In Skeeter Buster, some pupae mature earlier and some later, according to [4]. The first pupae start to mature when they reach cumulative physiological development *CD_t_* = 0.89. All of the pupae mature when they reach cumulative physiological development *CD_t_* =1.17. In between, only a fraction of the pupae mature based on equation 2 in [4]. In this case, a new mature pupal cohort is branched off of the original cohort with identical characteristics but mature. Note that this cohort is not processed again on the same day.

6. In CIMSiM, both male and female pupae obtain their weights from the larval cohort weight on the previous day. In Skeeter Buster, female pupae have a weight that is the average between the female larval weights on the previous day and on the current day. Male pupae inherit the weight of the male larvae on the previous day (to ensure that, even in optimal conditions, male pupae have a lower weight than female pupae).

7. In CIMSiM, females emerging from several different pupal cohorts on the same day are merged into one female adult cohort that is assigned an average weight. In Skeeter Buster, separate cohorts are created for females and males emerging from different pupal cohorts, with their own representative weights.

8. In CIMSiM, there is no variation in the fecundity of females in the same adult cohort. In Skeeter Buster, females emerging from a given pupal cohort are modeled individually, and their fecundity is calculated stochastically from a normal distribution, with an average value based on the weight of the female (see main text), and, based on Table 2 of [5], standard deviation equal to 0.3751946 multiplied by the mean fecundity.

9. In CIMSiM, the nominal daily survival of both male and female adults is 0.91 day^-1^. In Skeeter Buster, the nominal daily survival of male adults is 0.77 day^-1^, while the nominal daily survival of female adults is 0.89 day^-1^[6].

10. In CIMSiM, containers are divided into 2 cm layers for oviposition. In Skeeter Buster, containers are divided into 2 mm layers for oviposition.

11. In CIMSiM, if the water level is at the top layer of the container, eggs are only laid into the top layer. If the water level is lower than that, eggs are distributed evenly at the water level layer and one layer above. In Skeeter Buster, if the water level is at the top layer of the container, eggs are only laid into the top layer. If the water level is lower than that, eggs are distributed evenly into the layers between the water level layer and all the layers up to the top layer of the container, but at most into 19 layers above the water level.

12. In CIMSiM, the pupation weight threshold is calculated at the beginning of the day. In Skeeter Buster, it is calculated at the end of the day, right before pupation.

13. In CIMSiM, adult males are discarded. In Skeeter Buster, male and female adults are both retained and tracked.

14. In CIMSiM, the initial weight of larvae at hatch is 0.0034 mg. In Skeeter Buster, the initial weight of larvae at hatch is 0.001 mg, based on [7]. The minimal weight for larval survival is lowered to 0.0009 mg in Skeeter Buster.

15. In CIMSiM, fasting survival is not recalculated if the larval cohort is about to pupate on the given day, and the fasting survival calculated on the previous day is used. In Skeeter Buster, fasting survival is always recalculated.

16. In CIMSiM, the average lipid reserve of a larval cohort is always reset to its original value when starvation ends, and recalculated when starvation begins. In Skeeter Buster, it is recalculated when starvation begins and do not implement the unnecessary reset.

**References:**

1. Barbosa P, Peters TM, Greenough NC (1972) Overcrowding of mosquito populations: Responses of larval *Aedes aegypti* to stress. Environ Entomol 1: 89-93.

2. Chambers GM, Klowden MJ (1990) Correlation of nutritional reserves with a critical weight for pupation in larval *Aedes aegypti* mosquitos. J Am Mosq Control Assoc 6: 394-399.

3. Styer LM, Carey JR, Wang JL, Scott TW (2007) Mosquitoes do senesce: Departure from the paradigm of constant mortality. Am J Trop Med Hyg 76: 111-117.

4. Rueda LM, Patel KJ, Axtell RC, Stinner RE (1990) Temperature-dependent development and survival rates of *Culex quinquefasciatus*  and *Aedes aegypti* (Diptera: Culicidae). J Med Entomol 27: 892-898.

5. Naksathit AT, Scott TW (1998) Effects of female size on fecundity and survivorship of *Aedes aegypti* fed only human blood versus human blood plus sugar. J Am Mosquito Contr 14: 148-152.

6. McDonald PT (1977) Population characteristics of domestic *Aedes aegypti* (Diptera: Culicidae) in villages on Kenya coast. 1. Adult survival and population size. J Med Entomol 14: 42-48.

7. Gilpin ME, McClelland GAH (1979) Systems-analysis of the yellow fever mosquito *Aedes aegypti*. Forts Zool 25: 355-388.
